# Regularity of SLE in $(t,\kappa )$ and refined GRR estimates

**DOI:** 10.1007/s00440-021-01058-0

**Published:** 2021-05-06

**Authors:** Peter K. Friz, Huy Tran, Yizheng Yuan

**Affiliations:** 1 TU and WIAS, Berlin, Germany; 2 TU Berlin, Berlin, Germany

**Keywords:** 30C20, 60G17, 60G60, 60J67, 60K35

## Abstract

Schramm–Loewner evolution ($\hbox {SLE}_\kappa$) is classically studied via Loewner evolution with half-plane capacity parametrization, driven by $\sqrt{\kappa }$ times Brownian motion. This yields a (half-plane) valued random field $\gamma = \gamma (t, \kappa ; \omega )$. (Hölder) regularity of in $\gamma (\cdot ,\kappa ;\omega$), a.k.a. SLE trace, has been considered by many authors, starting with Rohde and Schramm (Ann Math (2) 161(2):883–924, 2005). Subsequently, Johansson Viklund et al. (Probab Theory Relat Fields 159(3–4):413–433, 2014) showed a.s. Hölder continuity of this random field for $\kappa < 8(2-\sqrt{3})$. In this paper, we improve their result to joint Hölder continuity up to $\kappa < 8/3$. Moreover, we show that the SLE$_\kappa$ trace $\gamma (\cdot ,\kappa )$ (as a continuous path) is stochastically continuous in $\kappa$ at all $\kappa \ne 8$. Our proofs rely on a novel variation of the Garsia–Rodemich–Rumsey inequality, which is of independent interest.

## Introduction

Schramm–Loewner evolution (SLE) is a random (non-self-crossing) path connecting two boundary points of a domain. To be more precise, it is a family of such random paths indexed by a parameter $\kappa \ge 0$. It has been first introduced by [19] to describe several random models from statistical physics. Since then, many authors have intensely studied this random object. Many connections to discrete processes and other geometric objects have been made, and nowadays SLE is one of the key objects in modern probability theory.

The typical way of constructing SLE is via the Loewner differential equation (see Sect. 3) which provides a correspondence between real-valued functions (“driving functions”) and certain growing families of sets (“hulls”) in a planar domain. For many (in particular more regular) driving functions, the growing families of hulls (or their boundaries) are continuous curves called traces. For Brownian motion, it is a non-trivial fact that for fixed $\kappa \ge 0$, the driving function $\sqrt{\kappa }B$ almost surely generates a continuous trace which we call SLE$_\kappa$ trace (see [16, 18]).

There has been a series of papers investigating the analytic properties of SLE, such as (Hölder and *p*-variation) regularity of the trace [5, 9, 15, 18]. See also [4, 20] for some recent attempts to understand better the existence of SLE trace.

A natural question is whether the SLE$_\kappa$ trace obtained from this construction varies continuously in the parameter $\kappa$. Another natural question is whether with probability 1 the construction produces a continuous trace simultaneously for all $\kappa \ge 0$. These questions have been studied in [10] where the authors showed that with probability 1, the SLE$_\kappa$ trace exists and is continuous in the range $\kappa \in [0,8(2-\sqrt{3})[$. In our paper we improve their result and extend it to $\kappa \in [0,8/3[$. (In fact, our result is a bit stronger than the following statement, see Theorems 3.2 and 4.1.)

### Theorem 1.1

Let *B* be a standard Brownian motion. Then almost surely the SLE$_\kappa$ trace $\gamma ^\kappa$ driven by $\sqrt{\kappa }B_t$, $t \in [0,1]$, exists for all $\kappa \in [0,8/3[$, and the trace (parametrised by half-plane capacity) is continuous in $\kappa \in [0,8/3[$ with respect to the supremum distance on [0, 1].

Stability of SLE trace was also recently studied in [12, Theorem 1.10]. They show the law of $\gamma ^{\kappa _n} \in C([0,1],{\mathbb {H}})$ converges weakly to the law of $\gamma ^{\kappa }$ in the topology of uniform convergence, whenever $\kappa _n \rightarrow \kappa < 8$. Of course, we get this as a trivial corollary of Theorem 1.1 in case of $\kappa < 8/3$. Our Theorem 1.2 (proved in Sect. 3.2) strengthens [12, Theorem 1.10] in three ways:

(i) we allow for any $\kappa \ne 8$;
(ii) we improve weak convergence to convergence in probability;
(iii) we strengthen convergence in $C([0,1],{\mathbb {H}})$ with uniform topology to $C^{p\text {-var}}([0,1],{\mathbb {H}})$ with optimal (cf. [5]) *p*-variation parameter, i.e. any $p > (1 + \kappa /8) \wedge 2$. The analogous statement for $\alpha$-Hölder topologies, $\alpha < \left( 1-\frac{\kappa }{24+2\kappa -8\sqrt{8+\kappa }}\right) \wedge \frac{1}{2}$, is also true.

Here and below we write $\Vert f \Vert ^p_{p\text {-var};[a,b]} \mathrel {\mathop :}=\sup \sum _{[s,t]\in \pi } |f(t)-f(s)|^p$, with $\sup$ taken over all partitions $\pi$ of [*a*, *b*]. The following theorem will be proved as Corollary 3.12.

### Theorem 1.2

Let *B* be a standard Brownian motion, and $\gamma ^\kappa$ the SLE$_\kappa$ trace driven by $\sqrt{\kappa }B_t$, $t \in [0,1]$, (and parametrised by half-plane capacity). For any $\kappa > 0$, $\kappa \ne 8$ and any sequence $\kappa _n \rightarrow \kappa$ we then have $\Vert \gamma ^\kappa -\gamma ^{\kappa _n}\Vert _{p\text {-var};[0,1]} \rightarrow 0$ in probability, for any $p > (1 + \kappa / 8) \wedge 2$.

There are two major new ingredients to our proofs. First, we prove in Sect. 5 a refined moment estimate for SLE increments in $\kappa$, improving upon [10]. Using standard notation [14, 18], for $\kappa > 0$, we denote by $(g^\kappa _t)_{t \ge 0}$ the forward SLE flow driven by $\sqrt{\kappa }B$, $j=1,2$, and by ${\hat{f}}^\kappa _t = (g^\kappa _t)^{-1}( \cdot +\sqrt{\kappa }B_t)$ the recentred inverse flow, also defined in Sect. 3 below.

Write $a \lesssim b$ for $a \le Cb$, with suitable constant $C<\infty$. The improved estimate (Proposition 3.5) reads

1 $$\begin{aligned} {\mathbb {E}}|{\hat{f}}^\kappa _t(i\delta )-{\hat{f}}^{{\tilde{\kappa }}}_t(i\delta )|^p \lesssim |\sqrt{\kappa }-\sqrt{{\tilde{\kappa }}}|^p \end{aligned}$$

for $1 \le p < 1+\frac{8}{\kappa }$. The interest in this estimate is when *p* is close to $1 + 8/\kappa$. No such estimate can be extracted from [10], as we explain in some more detail in Remark 3.6 below.

Secondly, our way of exploiting moment estimates such as (1) is fundamentally different in comparison with the Whitney-type partition technique of “$(t,y,\kappa )$”-space [10] (already seen in [18] without $\kappa$), combined with a Borel–Cantelli argument. Our key tool here is a new higher-dimensional variant of the Garsia–Rodemich–Rumsey (GRR) inequality [7] which is useful in its own right, essentially whenever one deals with random fields with very “different”—in our case *t* and $\kappa$—variables. The GRR inequality has been a useful tool in stochastic analysis to pass from moment bounds for stochastic processes to almost sure estimates of their regularity.

Let us briefly discuss the existing (higher-dimensional) GRR estimates (e.g. [21, Exercise 2.4.1], [1, 3, 8]) and their shortcomings in our setting. When we try to apply one of these versions to SLE (as a two-parameter random field in $(t,\kappa )$), we wish to estimate moments of $|\gamma (t,\kappa )-\gamma (s,{\tilde{\kappa }})|$, where we denote the SLE$_\kappa$ trace by $\gamma (\cdot ,\kappa )$. In [5], the estimate

$$\begin{aligned} {\mathbb {E}}|\gamma (t,\kappa )-\gamma (s,\kappa )|^\lambda \lesssim |t-s|^{(\lambda +\zeta )/2} \end{aligned}$$

with suitable $\lambda >1$ and $\zeta$ has been given. We will show in Proposition 3.3 that

$$\begin{aligned} {\mathbb {E}}|\gamma (s,\kappa )-\gamma (s,{\tilde{\kappa }})|^p \lesssim |\kappa -{\tilde{\kappa }}|^p \end{aligned}$$

for suitable $p>1$. Applying this estimate with $p=\lambda$, we obtain an estimate for ${\mathbb {E}}|\gamma (t,\kappa )-\gamma (s,{\tilde{\kappa }})|^\lambda$, and can apply a GRR lemma from [1, 3]. The condition for applying it is $((\lambda +\zeta )/2)^{-1}+p^{-1} = ((\lambda +\zeta )/2)^{-1}+\lambda ^{-1} < 1$. But in doing so, we do not use the best estimates available to us. That is, the above estimate typically holds for some $p > \lambda$. On the other hand, we can only estimate the $\lambda$-th moment (and no higher ones) of $|\gamma (t,\kappa )-\gamma (s,\kappa )|$. This asks for a version of the GRR lemma that respects distinct exponents in the available estimates, and is applicable when $((\lambda +\zeta )/2)^{-1}+p^{-1} < 1$ with $p > \lambda$ (a weaker condition than above).

We are going to prove the following refined GRR estimates in two dimensions, as required by our application, noting that extension to higher dimension follow the same argument.

### Lemma 1.3

Let *G* be a continuous function (defined on some rectangle) such that, for some integers $J_1, J_2$,

$$\begin{aligned} |G(x_1,x_2)-G(y_1,y_2)|&\le |G(x_1,x_2)-G(y_1,x_2)| + |G(y_1,x_2)-G(y_1,y_2)|\\&\le \sum ^{J_1}_{j=1} |A_{1j}(x_1,y_1;x_2)| + \sum ^{J_1}_{j=1} |A_{2j}(y_1;x_2,y_2)|. \end{aligned}$$

Suppose that for all *j*,

$$\begin{aligned} \iiint \frac{|A_{1j}(u_1,v_1;u_2)|^{q_{1j}}}{|u_1-v_1|^{\beta _{1j}}} \, du_1 \, dv_1 \, du_2&< \infty ,\\ \iiint \frac{|A_{2j}(v_1;u_2,v_2)|^{q_{2j}}}{|u_2-v_2|^{\beta _{2j}}} \, dv_1 \, du_2 \, dv_2&< \infty . \end{aligned}$$

Then, under suitable conditions on the exponents,

$$\begin{aligned} |G(x_1,x_2)-G(y_1,y_2)| \lesssim |x_1-y_1|^{\gamma ^{(1)}} +|x_2-y_2|^{\gamma ^{(2)}}. \end{aligned}$$

Observe that the exponents $q_{1j}, q_{2j}$ are allowed to vary, exactly as required for our application to SLE. We also note that the flexibility to have $J_1,J_2 >1$ is used in the proof of Theorem 1.2 but not 1.1.

One might ask whether one can further improve Theorem 1.1 to all $\kappa \ge 0$. With the methods of this paper, it would require a better moment estimate in the style of (1) with larger exponent on the right-hand side. If such an estimate were to hold true with arbitrarily large exponent on the right-hand side (and any suitable exponent on the left-hand side), which is not clear to us, almost sure continuity of the random field in all $(t,\kappa )$ with $\kappa \ne 8$ would follow.

## A Garsia–Rodemich–Rumsey lemma with mixed exponents

In this section we prove a variant of the Garsia–Rodemich–Rumsey inequality and Kolmogorov’s continuity theorem. The classical Kolmogorov’s theorem goes by a “chaining” argument (see e.g. [13, Theorem 1.4.1] or [23, Appendix A.2]), but can also be obtained from the GRR inequality (see e.g. [21, Corollary 2.1.5]). In the case of proving Hölder continuity of processes, the GRR approach provides more powerful statements (cf. [6, Appendix A]). In particular, we obtain bounds on the Hölder constant of the process that are more informative and easier to manipulate, which will be useful in the proof of Theorem 4.1. (Although there are drawbacks of the GRR approach when generalising to more refined modulus of continuity, see the discussion in [23, Appendix A.4].)

We discuss some of the extensive literature that deal with the generality of GRR and Kolmogorov’s theorem. The reader may skip this discussion and continue straight with the results of this section.

There are some direct generalisations of GRR and Kolmogorov’s theorem to higher dimensions, e.g. [21, Exercise 2.4.1], [13, Theorem 1.4.1], [1, 3, 8]. Moreover, there have been more systematic studies in a general setting under the titles metric entropy bounds and majorising measures. They derive bounds and path continuity of stochastic processes mainly from the structure of certain pseudometrics that the processes induce on the parameter space, such as $d_X(s,t) \mathrel {\mathop :}=({\mathbb {E}}|X(s)-X(t)|^2)^{1/2}$. A large amount of the theory is found in the book by Talagrand [23]. These results due to, among others, R. M. Dudley, N. Kôno, X. Fernique, M. Talagrand, and W. Bednorz. Their main purpose is to allow different structures of the parameter space and inhomogeneity of the stochastic process (see e.g. [2, 11, 23]).

We explain why the existing results do not cover the adaption that we are seeking in this section. The general idea for applying the theory of metric entropy bounds would be considering the metric $d_X(s,t) = ({\mathbb {E}}|X(s)-X(t)|^{q})^{1/q}$ for some $q>1$.

Let us consider a random process defined on the parameter space $T = [0,1]^2$ that satisfies

2 $$\begin{aligned} \begin{aligned} {\mathbb {E}}|X(s_1,s_2)-X(t_1,s_2)|^{q_1}&\le |s_1-t_1|^{\alpha _1},\\ {\mathbb {E}}|X(t_1,s_2)-X(t_1,t_2)|^{q_2}&\le |s_2-t_2|^{\alpha _2}, \end{aligned} \end{aligned}$$

where $q_1$ and $q_2$ might be different, say $q_1 < q_2$. By Hölder’s inequality,

3 $$\begin{aligned} {\mathbb {E}}|X(t_1,s_2)-X(t_1,t_2)|^{q_1} \le \left( {\mathbb {E}}|X(t_1,s_2)-X(t_1,t_2)|^{q_2} \right) ^{q_1/q_2}. \end{aligned}$$

Write $t = (t_1,t_2)$, $s = (s_1,s_2)$. We may let

$$\begin{aligned} ({\mathbb {E}}|X(s)-X(t)|^{q})^{1/q} \le |s_1-t_1|^{\alpha _1/q_1}+|s_2-t_2|^{\alpha _2/q_2} =\mathrel {\mathop :}|||s-t||| =\mathrel {\mathop :}d(s,t) \end{aligned}$$

where we can take $q = q_1$ (but not $q=q_2$ without knowing any bounds on higher moments of $|X(s_1,s_2)-X(t_1,s_2)|$).

We explain now that we have already lost some sharpness when we estimated (3) using Hölder’s inequality. Indeed, all the results [11, Theorem 3], [23, (13.141)], [23, Theorem B.2.4], [2, Corollary 1] are based on finding an increasing convex function $\varphi$ such that

4 $$\begin{aligned} {\mathbb {E}}\varphi \left( \frac{|X(s)-X(t)|}{d(s,t)}\right) \le 1. \end{aligned}$$

Observe that we can take $\varphi (x) = x^{q_1}$ at best. To apply any of these results, the condition turns out to be $\frac{1}{\alpha _1}+\frac{q_2}{q_1\alpha _2} < 1$. In fact, [23, Theorem 13.5.8] implies that we cannot expect anything better just from the assumption (4). More precisely, the theorem states that in general, when we assume only (4), in order to deduce any pathwise bounds for the process *X*, we need to have

$$\begin{aligned} \int _0^\delta \varphi ^{-1}\left( \frac{1}{\mu (B(t,\varepsilon ))}\right) \, d\varepsilon < \infty , \end{aligned}$$

with *B* denoting the ball with respect to the metric *d*, and $\mu$ e.g. the Lebesgue measure. In our setup this turns out to the condition $\frac{1}{\alpha _1}+\frac{q_2}{q_1\alpha _2} < 1$.

We will show in Theorem 2.8 that by using the condition (2) instead of (4), we can relax this condition to $\frac{1}{\alpha _1}+\frac{1}{\alpha _2} < 1$. In case $\frac{1}{\alpha _1}+\frac{1}{\alpha _2}< 1 < \frac{1}{\alpha _1}+\frac{q_2}{q_1\alpha _2}$, this is an improvement. We have not found this possibility in any of the existing references.

We now turn to our version of the Garsia–Rodemich–Rumsey inequality that allows us to make use of different exponents $q_1 \ne q_2$. In addition to the scenario (2), we allow also the situation when e.g. $|X(s_1,s_2)-X(t_1,s_2)| \le A_{11}+A_{12}$ with ${\mathbb {E}}|A_{1j}|^{q_{1j}} \le |s_1-t_1|^{\alpha _{1j}}$ for some $q_{1j},\alpha _{1j}$, $j=1,2$, where possibility $q_{11} \ne q_{12}$.

Let (*E*, *d*) be a metric space. We can assume *E* to be isometrically embedded in some larger Banach space (by the Kuratowski embedding). To ease the notation, we write $|x-y| = d(x,y)$ both for the distance in *E* and for the distance in ${\mathbb {R}}$. For a Borel set *A* we denote by $|A|$ its Lebesgue measure and $\fint _A f = \frac{1}{|A|} \int _A f$.

In what follows, let $I_1$ and $I_2$ be two (either open or closed) non-trivial intervals of $\mathbb {R}$.

### Lemma 2.1

Let $G \in C(I_1\times I_2)$ be a continuous function, with values in a metric space *E*, such that

5 $$\begin{aligned} |G(x_1,x_2)-G(y_1,y_2) | \le \sum ^{J_1}_{j=1} |A_{1j}(x_1,y_1;x_2)| + \sum ^{J_2}_{j=1} |A_{2j}(y_1;x_2,y_2)| \end{aligned}$$

for all $(x_1,x_2), (y_1,y_2) \in I_1 \times I_2$, where $A_{1j}{:}\,I_1 \times I_1 \times I_2\rightarrow \mathbb {R}$, $1\le j\le J_1$, $A_{2j}{:}\, I_1 \times I_2 \times I_2\rightarrow \mathbb {R}$, $1\le j\le J_2$, are measurable functions. Suppose that

6 $$\begin{aligned} \iiint _{I_1 \times I_1 \times I_2} \frac{|A_{1j}(u_1,v_1;u_2)|^{q_{1j}}}{|u_1-v_1|^{\beta _{1j}}} \, du_1 \, dv_1 \, du_2&\le M_{1j}, \end{aligned}$$

7 $$\begin{aligned} \iiint _{I_1 \times I_2 \times I_2} \frac{|A_{2j}(v_1;u_2,v_2)|^{q_{2j}}}{|u_2-v_2|^{\beta _{2j}}} \, dv_1 \, du_2 \, dv_2&\le M_{2j} \end{aligned}$$

for all *j*, where $q_{ij} \ge 1$, $\beta _i := \min _j \beta _{ij} > 2$, $i=1,2$, and $(\beta _1-2)(\beta _2-2)-1 > 0$. Fix any $a,b > 0$. Then

8 $$\begin{aligned}&|G(x_1,x_2)-G(y_1,y_2)| \le C \sum _j M_{1j}^{1/q_{1j}} \, \left( |x_1-y_1|^{\gamma ^{(1)}_{1j}}+|x_2 -y_2|^{\gamma ^{(2)}_{1j}}\right) \nonumber \\&\quad +\,C \sum _j M_{2j}^{1/q_{2j}} \, \left( |x_1-y_1|^{\gamma ^{(1)}_{2j}}+|x_2-y_2|^{\gamma ^{(2)}_{2j}}\right) \end{aligned}$$

for all $(x_1,x_2), (y_1,y_2) \in I_1 \times I_2$, where $\gamma ^{(1)}_{1j} = \dfrac{\beta _{1j}-2-b}{q_{1j}}$, $\gamma ^{(2)}_{1j} = \dfrac{(\beta _{1j}-2)a-1}{q_{1j}}$, $\gamma ^{(1)}_{2j} = \dfrac{(\beta _{2j}-2)b-1}{q_{2j}}$, $\gamma ^{(2)}_{2j} = \dfrac{\beta _{2j}-2-a}{q_{2j}}$, and $C < \infty$ is a constant that depends on $(q_{ij}),(\beta _{ij}),a,b,|I_1|,|I_2|$.

### Remark 2.2

The statement is already true when $q_{ij}>0$ (not necessarily $\ge 1$) and can be shown by an argument similarly as in [21, Theorem 2.1.3 and Exercise 2.4.1]. We have decided to stick to $q_{ij} \ge 1$ since the proof is simpler here.

### Proof

Note that for any continuous function *G* and a sequence $B_n$ of sets with ${\text {diam}}(\{x\} \cup B_n) \rightarrow 0$ we have $G(x) = \lim _n \fint _{B_n} G$. (Recall that we can view *E* as a subspace of some Banach space, so that the integral is well-defined.)

Let $(x_1,x_2), (y_1,y_2) \in I_1 \times I_2$. Using the above observation, we will approximate $G(x_1,x_2)$ and $G(y_1,y_2)$ by well-chosen sequences of sets.

We pick a sequence of rectangles $I^n_1 \times I^n_2 \subseteq I_1 \times I_2$, $n \ge 0$, with the following properties:

- $$(x_1,x_2), (y_1,y_2) \in I^0_1 \times I^0_2$$.
- $$(x_1,x_2) \in I^n_1 \times I^n_2$$ for all *n*.
- $$|I^n_i| = R_i^{-n} d_i$$, $$i=1,2$$, with parameters
  $$\begin{aligned} R_1,R_2> 1,\quad d_1,d_2 > 0 \end{aligned}$$
  chosen later.

In order for such a sequence of rectangles to exist, we must have

$$\begin{aligned} |x_i-y_i| \le d_i \le |I_i|, \quad i=1,2, \end{aligned}$$

since we require $x_i,y_i \in I^0_i \subseteq I_i$. Conversely, this condition guarantees the existence of such a sequence.

We will bound

$$\begin{aligned} \left|{G(x_1,x_2) - \fint \fint _{I^0_1 \times I^0_2} G}\right|\le \sum _{n \in {\mathbb {N}}} \left|{\fint \fint _{I^n_1 \times I^n_2} G - \fint \fint _{I^{n-1}_1 \times I^{n-1}_2} G}\right|. \end{aligned}$$

The same argument applies also to $G(y_1,y_2)$ where we can pick the same initial rectangle $I^0_1 \times I^0_2$. Hence, this will give us a bound on $|G(x_1,x_2)-G(y_1,y_2)|$.

By the assumption (5) we have

$$\begin{aligned} \begin{aligned}&\left|{\fint \fint _{I^n_1 \times I^n_2} G - \fint \fint _{I^{n-1}_1 \times I^{n-1}_2} G}\right|\\&\quad = \left|{\fint \fint _{I^n_1 \times I^n_2}\fint \fint _{I^{n-1}_1 \times I^{n-1}_2} (G(u_1,u_2)-G(v_1,v_2)) \,du_1\,du_2\,dv_1\,dv_2}\right|\\&\quad \le \sum _j \fint _{I^n_1}\fint _{I^{n-1}_1}\fint _{I^n_2} |A_{1j}(u_1,v_1;u_2)| + \sum _j\fint _{I^{n-1}_1}\fint _{I^n_2}\fint _{I^{n-1}_2} |A_{2j}(v_1;u_2,v_2)|. \end{aligned} \end{aligned}$$

Recall that $|I^n_i| = R_i^{-n}d_i$ and that $|u_i-v_i| \le C R_i^{-n}d_i$ for any $u_i \in I^n_i$, $v_i \in I^{n-1}_i$. This and Hölder’s inequality imply

$$\begin{aligned} \begin{aligned}&\fint _{I^n_1}\fint _{I^{n-1}_1}\fint _{I^n_2} |A_{1j}(u_1,v_1;u_2)|\\&\quad \le C (R_1^{-n}d_1)^{\beta _{1j}/q_{1j}} \fint _{I^n_1} \fint _{I^{n-1}_1}\fint _{I^n_2} \frac{|A_{1j}(u_1,v_1;u_2)|}{|u_1-v_1|^{\beta _{1j}/q_{1j}}}\\&\quad \le C (R_1^{-n}d_1)^{\beta _{1j}/q_{1j}} \left( \fint _{I^n_1}\fint _{I^{n-1}_1}\fint _{I^n_2} \frac{ |A_{1j}(u_1,v_1;u_2)|^{q_{1j}}}{|u_1-v_1|^{\beta _{1j}}} \right) ^{1/q_{1j}}\\&\quad \le C (R_1^{-n}d_1)^{\beta _{1j}/q_{1j}} \left( (R_1^{-n}d_1)^{-2} (R_2^{-n}d_2)^{-1} M_{1j} \right) ^{1/q_{1j}} \\&\quad = C \left( (R_1^{-n}d_1)^{\beta _{1j}-2} (R_2^{-n}d_2)^{-1} M_{1j} \right) ^{1/q_{1j}}. \end{aligned} \end{aligned}$$

Similarly,

$$\begin{aligned} \fint _{I^{n-1}_1}\fint _{I^n_2}\fint _{I^{n-1}_2}|A_{2j}(v_1;u_2,v_2)| \le C \left( (R_2^{-n}d_2)^{\beta _{2j}-2} (R_1^{-n}d_1)^{-1} M_{2j} \right) ^{1/q_{2j}}. \end{aligned}$$

We want to sum the above expressions for all *n*, which is possible if and only if both $R_1^{\beta _{1j}-2} R_2^{-1} > 1$ and $R_2^{\beta _{2j}-2} R_1^{-1} > 1$. The best pick is $R_2 = R_1^{\frac{\beta _1-1}{\beta _2-1}}$ (the exact scale of $R_1$ does not matter), and the condition becomes $(\beta _1-2)(\beta _2-2)-1 > 0$ (assuming $\beta _1,\beta _2 > 2$). In that case, we finally get

9 $$\begin{aligned}&|G(x_1,x_2)-G(y_1,y_2)| \nonumber \\&\quad \le C \sum _j \left( d_1^{\beta _{1j}-2} d_2^{-1} M_{1j} \right) ^{1/q_{1j}} + C \sum _j \left( d_2^{\beta _{2j}-2} d_1^{-1} M_{2j} \right) ^{1/q_{2j}} \end{aligned}$$

It remains to pick $d_1,d_2 > 0$. Let $d_1 := |x_1-y_1| \vee |x_2-y_2|^a$, $d_2 := |x_1-y_1|^b \vee |x_2-y_2|$, and suppose for the moment that $d_1 \le |I_1|$, $d_2 \le |I_2|$. (The conditions $d_1 \ge |x_1 - y_1|$, $d_2 \ge |x_2 - y_2|$ are satisfied by our choice.). In this case the inequality (9) becomes

10 $$\begin{aligned} \begin{aligned}&|G(x_1,x_2)-G(y_1,y_2)| \\&\quad \le C \sum _j M_{1j}^{1/q_{1j}} \, \left( |x_1-y_1|^{\beta _{1j}-2-b}+|x_2-y_2|^{(\beta _{1j}-2)a-1}\right) ^{1/q_{1j}}\\&\qquad +\,C \sum _j M_{2j}^{1/q_{2j}} \, \left( |x_1-y_1|^{(\beta _{2j}-2)b-1}+|x_2-y_2|^{\beta _{2j}-2-a}\right) ^{1/q_{2j}} .\end{aligned} \end{aligned}$$

This proves the claim in case $d_1 \le |I_1|$, $d_2 \le |I_2|$.

It remains to handle the case when $d_1 > |I_1|$ or $d_2 > |I_2|$. In that case we pick ${\hat{d}}_1 = d_1 \wedge |I_1|$ and ${\hat{d}}_2 = d_2 \wedge |I_2|$ instead of $d_1$ and $d_2$. The conditions $|x_1-y_1| \le {\hat{d}}_1 \le |I_1|$ and $|x_2-y_2| \le {\hat{d}}_2 \le |I_2|$ are now satisfied, and in (9), we instead have

11 $$\begin{aligned} \begin{aligned} {\hat{d}}_1^{\beta _{1j}-2} {\hat{d}}_2^{-1}&\le \frac{d_2}{d_2 \wedge |I_2|} \ d_1^{\beta _{1j}-2} d_2^{-1} = \left( \frac{|x_1-y_1|^b}{|I_2|} \vee 1 \right) d_1^{\beta _{1j}-2} d_2^{-1},\\ {\hat{d}}_1^{-1} {\hat{d}}_2^{\beta _{2j}-2}&\le \frac{d_1}{d_1 \wedge |I_1|} \ d_1^{-1} d_2^{\beta _{2j}-2} = \left( \frac{|x_2-y_2|^a}{|I_1|} \vee 1 \right) d_1^{-1} d_2^{\beta _{2j}-2}, \end{aligned} \end{aligned}$$

i.e. the same result (10) holds with the additional constants $\left( \frac{|x_1-y_1|^b}{|I_2|} \vee 1 \right)$ and $\left( \frac{|x_2-y_2|^a}{|I_1|} \vee 1 \right)$ (which can be bounded by a constant depending on $a,b,|I_1|,|I_2|$ since $a,b \ge 0$). $\square$

### Remark 2.3

The dependence of the multiplicative constant *C* on $|I_1|$ and $|I_2|$ is specified in (11). This can be convenient when we want to apply the lemma to different domains.

A more accurate version is

$$\begin{aligned} {\hat{d}}_1^{\beta _{1j}-2} {\hat{d}}_2^{-1}&= \left( \frac{d_1 \wedge |I_1|}{d_1} \right) ^{\beta _{1j}-2} \frac{d_2}{d_2 \wedge |I_2|} \ d_1^{\beta _{1j}-2} d_2^{-1}\\&= \left( \frac{|I_1|}{|x_2-y_2|^a} \wedge 1 \right) ^{\beta _{1j}-2} \left( \frac{|x_1-y_1|^b}{|I_2|} \vee 1 \right) d_1^{\beta _{1j}-2} d_2^{-1},\\ {\hat{d}}_1^{-1} {\hat{d}}_2^{\beta _{2j}-2}&= \left( \frac{d_2 \wedge |I_2|}{d_2} \right) ^{\beta _{2j}-2} \frac{d_1}{d_1 \wedge |I_1|} \ d_1^{-1} d_2^{\beta _{2j}-2}\\&= \left( \frac{|I_2|}{|x_1-y_1|^b} \wedge 1 \right) ^{\beta _{2j}-2} \left( \frac{|x_2-y_2|^a}{|I_1|} \vee 1 \right) d_1^{-1} d_2^{\beta _{2j}-2}. \end{aligned}$$

### Remark 2.4

We could have added some more flexibility by allowing the exponents $(q_{ij}),(\beta _{ij})$ to vary with $u_1,u_2$, but again we will not need it for our result.

### Remark 2.5

We have a free choice of $a,b \ge 0$ which affects the Hölder exponents $\gamma ^{(1)}_{ij}, \gamma ^{(2)}_{ij}$. In general, it is not simple to spell out the optimal choice of *a*, *b* and hence the optimal Hölder exponents. Usually we are interested in the overall exponents (i.e. $\min _{i,j} \gamma ^{(1)}_{ij}$, $\min _{i,j} \gamma ^{(2)}_{ij}$), and we can solve

$$\begin{aligned} \min _j \gamma ^{(1)}_{1j}&= \min _j \gamma ^{(1)}_{2j},\\ \min _j \gamma ^{(2)}_{1j}&= \min _j \gamma ^{(2)}_{2j} \end{aligned}$$

to find the optimal choice for *a*, *b*.

For instance, in case $\beta _{1j} = \beta _1$ and $\beta _{2j} = \beta _2$ for all *j*, the best choice is

$$\begin{aligned} a = \frac{q_1(\beta _2-2)+q_2}{q_2(\beta _1-2)+q_1},\quad b = \frac{q_2(\beta _1-2)+q_1}{q_1(\beta _2-2)+q_2}, \end{aligned}$$

resulting in

$$\begin{aligned} \gamma ^{(1)} = \frac{(\beta _1-2)(\beta _2-2)-1}{q_1(\beta _2-2)+q_2},\quad \gamma ^{(2)} = \frac{(\beta _1-2)(\beta _2-2)-1}{q_2(\beta _1-2)+q_1} \end{aligned}$$

where $$q_i = \max _j q_{ij}$$.

In general, we could choose $a = \frac{\beta _2-1}{\beta _1-1}$, $b=\frac{\beta _1-1}{\beta _2-1}$, resulting in

$$\begin{aligned} \gamma ^{(1)}_{1j}&= \frac{(\beta _{1j}-2)(\beta _2-2)-1+\beta _{1j}-\beta _1}{q_{1j}(\beta _2-1)},\quad&\gamma ^{(2)}_{1j}&= \frac{(\beta _{1j}-2)(\beta _2-2)-1+\beta _{1j}-\beta _1}{q_{1j}(\beta _1-1)},\\ \gamma ^{(1)}_{2j}&= \frac{(\beta _1-2)(\beta _{2j}-2)-1+\beta _{2j}-\beta _2}{q_{2j}(\beta _2-1)},\quad&\gamma ^{(2)}_{2j}&= \frac{(\beta _1-2)(\beta _{2j}-2)-1+\beta _{2j}-\beta _2}{q_{2j}(\beta _1-1)}. \end{aligned}$$

But this is not necessarily the optimal choice.

### Remark 2.6

Notice that the condition to apply the lemma does only depend on $(\beta _{ij})$, not $(q_{ij})$, but the resulting Hölder-exponents will.

### Remark 2.7

The proof straightforwardly generalises to higher dimensions.

Using our version of the GRR lemma, we can show another version of the Kolmogorov continuity condition. Here we suppose $I_1$, $I_2$ are *bounded* intervals.

### Theorem 2.8

Let *X* be a random field on $I_1 \times I_2$ taking values in a separable Banach space. Suppose that, for $(x_1,x_2), (y_1,y_2) \in I_1 \times I_2$, we have

12 $$\begin{aligned} |X(x_1,x_2)-X(y_1,y_2)| \le \sum _{j=1}^{J_1} |A_{1j}(x_1,y_1;x_2)| + \sum _{j=1}^{J_2} |A_{2j}(y_1;x_2,y_2)| \end{aligned}$$

with measurable real-valued $A_{ij}$ that satisfy

13 $$\begin{aligned} \begin{aligned} {\mathbb {E}}|A_{1j}(x_1,y_1;x_2)|^{q_{1j}}&\le C' \, |x_1-y_1|^{\alpha _{1j}},\\ {\mathbb {E}}|A_{2j}(y_1;x_2,y_2)|^{q_{2j}}&\le C' \, |x_2-y_2|^{\alpha _{2j}} \end{aligned} \end{aligned}$$

with a constant $$C' < \infty $$.

Moreover, suppose $q_{ij} \ge 1$, $\alpha _i = \min _j \alpha _{ij} > 1$, $i=1,2$, and $\alpha _1^{-1}+\alpha _2^{-1} < 1$.

Then *X* has a Hölder-continuous modification ${\hat{X}}$. Moreover, for any

$$\begin{aligned} \gamma ^{(1)}< \frac{(\alpha _1-1)(\alpha _2-1)-1}{q_1(\alpha _2-1)+q_2},\quad \gamma ^{(2)} < \frac{(\alpha _1-1)(\alpha _2-1)-1}{q_2(\alpha _1-1)+q_1}, \end{aligned}$$

where $q_i = \max _j q_{ij}$, there is a random variable *C* such that

$$\begin{aligned} |{\hat{X}}(x_1,x_2)-{\hat{X}}(y_1,y_2)| \le C \left( |x_1-y_1|^{\gamma ^{(1)}}+|x_2-y_2|^{\gamma ^{(2)}}\right) \end{aligned}$$

and $${\mathbb {E}}[C^{q_{\text {min}}}] < \infty $$ for $$q_{\text {min}}=\min _{i,j} q_{ij}$$.

### Remark 2.9

In case $\alpha _{1j} = \alpha _1$ and $\alpha _{2j} = \alpha _2$ for all *j*, the expressions for the Hölder exponents $\gamma ^{(1)}, \gamma ^{(2)}$ given above are sharp. In the general case, the exponents may be improved, following an optimisation described in Remark 2.5.

### Remark 2.10

The constants $C'$ can be replaced by (deterministic) functions that are integrable in $(x_1, x_2)$, without change of the proof. But one would need to formulate the condition more carefully, therefore we decided to not include it.

We point out that in case $J_1=J_2=1$ and $q_1=q_2$, this agrees with the two-dimensional version of the (inhomogeneous) Kolmogorov criterion [13, Theorem 1.4.1].

### Proof

**Part 1.** Suppose first that *X* is already continuous. In that case we can directly apply Lemma 2.1. The expectation of the integrals (6) and (7) are finite if $\beta _{ij} < \alpha _{ij}+1$ for all *i*, *j*. By choosing $\beta _{ij}$ as large as possible, the conditions $(\beta _1-2)(\beta _2-2)-1 > 0$ and $\beta _1 > 2$, $\beta _2 > 2$ are satisfied if $\alpha _1^{-1}+\alpha _2^{-1} < 1$ and $\alpha _1 > 1$, $\alpha _2 > 1$.

Since the (random) constants $M_{ij}$ in Lemma 2.1 are almost surely finite, *X* is Hölder continuous as quantified in (8), and the Hölder constants $M_{ij}^{1/q_{ij}}$ have $q_{ij}$-th moments since they are just the integrals (6). The formulas for the Hölder exponents follow from the analysis in Remark 2.5.

**Part 2.** Now, suppose *X* is arbitrary. We need to construct a continuous version of *X*. It suffices to show that *X* is uniformly continuous on a dense set $D \subseteq I_1 \times I_2$. Indeed, we can then apply Doob’s separability theorem to obtain a separable (and hence continuous) version of *X*, or alternatively construct ${\hat{X}}$ by setting ${\hat{X}} = X$ on *D* and extend ${\hat{X}}$ continuously to $I_1 \times I_2$. Then ${\hat{X}}$ is a modification of *X* because they agree on a dense set *D* and are both stochastically continuous [as follows from (12) and (13)].

We use a standard argument that can be found e.g. in [22, pp. 8–9].

We can assume without loss of generality that $X({\bar{x}}_1,{\bar{x}}_2) = 0$ for some $({\bar{x}}_1,{\bar{x}}_2) \in I_1 \times I_2$ (otherwise just consider $Y(x_1,x_2) = X(x_1,x_2)-X({\bar{x}}_1,{\bar{x}}_2)$).

In particular, the conditions (12) and (13) imply that $X(x_1,x_2)$ is an integrable random variable with values in a separable Banach space for every $(x_1,x_2)$.

Fix any countable dense subset $D \subseteq I_1 \times I_2$. Let

$$\begin{aligned} {\mathcal {G}} \mathrel {\mathop :}=\sigma ( \{X(x_1,x_2) \mid (x_1,x_2) \in D\}). \end{aligned}$$

We can pick an increasing sequence of *finite* $\sigma$-algebras ${\mathcal {G}}_n$ such that ${\mathcal {G}} = \sigma \left( \bigcup _n {\mathcal {G}}_n\right)$. By martingale convergence, we have

$$\begin{aligned} X^{(n)}(x_1,x_2) \rightarrow X(x_1,x_2) \end{aligned}$$

almost surely for $(x_1,x_2) \in D$ where $X^{(n)}(x_1,x_2) \mathrel {\mathop :}={\mathbb {E}}[X(x_1,x_2) \mid {\mathcal {G}}_n]$.

Moreover, (12) implies

$$\begin{aligned} |X^{(n)}(x_1,x_2)-X^{(n)}(y_1,y_2)| \le \sum _{j=1}^{J_1} |A_{1j}^{(n)}(x_1,y_1;x_2)| + \sum _{j=1}^{J_2} |A_{2j}^{(n)}(y_1;x_2,y_2)| \end{aligned}$$

where $|A_{ij}^{(n)}(\ldots )| \mathrel {\mathop :}={\mathbb {E}}[|A_{ij}^{(n)}(...)| \mid {\mathcal {G}}_n]$. By Jensen’s inequality and (13), we have

$$\begin{aligned} \begin{aligned} {\mathbb {E}}|A_{1j}^{(n)}(x_1,y_1;x_2)|^{q_{1j}}&\le {\mathbb {E}}|A_{1j}(x_1,y_1;x_2)|^{q_{1j}} \le C' \, |x_1-y_1|^{\alpha _{1j}},\\ {\mathbb {E}}|A_{2j}^{(n)}(y_1;x_2,y_2)|^{q_{2j}}&\le {\mathbb {E}}|A_{2j}(y_1;x_2,y_2)|^{q_{2j}} \le C' \, |x_2-y_2|^{\alpha _{2j}}. \end{aligned} \end{aligned}$$

In particular, $X^{(n)}$ is stochastically continuous, and since ${\mathcal {G}}_n$ is finite, $X^{(n)}$ is almost surely continuous. Applying Lemma 2.1 yields

$$\begin{aligned}&|X^{(n)}(x_1,x_2)-X^{(n)}(y_1,y_2)| \le C \sum _j (M^{(n)}_{1j})^{1/q_{1j}} \, \left( |x_1-y_1 |^{\gamma ^{(1)}_{1j}}+|x_2-y_2|^{\gamma ^{(2)}_{1j}}\right) \\&\quad +\,C \sum _j (M^{(n)}_{2j})^{1/q_{2j}} \, \left( |x_1- y_1|^{\gamma ^{(1)}_{2j}}+|x_2-y_2|^{\gamma ^{(2)}_{2j}}\right) \end{aligned}$$

where $M^{(n)}_{ij}$ are defined as the integrals (6) and (7) with $A_{ij}^{(n)}$.

It follows that on *D* we have

$$\begin{aligned}&|X(x_1,x_2)-X(y_1,y_2)| \le C \sum _j {\tilde{M}}_{1j}^{1/q_{1j}} \, \left( |x_1-y_1|^{\gamma ^{(1)}_{1j}}+|x_2-y_2|^{\gamma ^{(2)}_{1j}}\right) \\&\quad +\,C \sum _j {\tilde{M}}_{2j}^{1/q_{2j}} \, \left( |x_1-y_1|^{\gamma ^{(1)}_{2j}}+|x_2-y_2|^{\gamma ^{(2)}_{2j}}\right) \end{aligned}$$

where ${\tilde{M}}_{ij} \mathrel {\mathop :}=\liminf _n M^{(n)}_{ij}$. By Fatou’s lemma,

$$\begin{aligned} {\mathbb {E}}{\tilde{M}}_{ij} \le \liminf _n {\mathbb {E}}M^{(n)}_{ij} < \infty , \end{aligned}$$

implying that ${\tilde{M}}_{ij} < \infty$, hence *X* is uniformly continuous on *D*. $\square$

One-dimensional variants of Lemma 2.1 and Theorem 2.8 can also be derived. Having shown the two-dimensional results Lemma 2.1 and Theorem 2.8, there is no need for an additional proof of their one-dimensional variants, since we can extend any one-parameter function *G* to a two-parameter function via ${\tilde{G}}(x_1,x_2) := G(x_1)$. This immediately implies the following results.

### Corollary 2.11

Let *G* be a continuous function on an interval *I* such that

$$\begin{aligned} |G(x)-G(y)| \le \sum _{j=1}^J |A_j(x,y)| \end{aligned}$$

for all $x,y \in I$, where $A_j{:}\,I \times I \rightarrow {\mathbb {R}}$, $j=1,\ldots ,J$, are measurable functions that satisfy

$$\begin{aligned} \iint _{I \times I} \frac{|A_j(u,v) |^{q_j}}{|u-v|^{\beta _j}} \, du \, dv \le M_j \end{aligned}$$

with some $q_j \ge 1$, $\beta _j > 2$. Then

$$\begin{aligned} |G(x)-G(y)| \le C \sum _j M_j^{1/q_j} |x-y|^{\gamma _j} \end{aligned}$$

for all $x,y \in I$, where $\gamma _j = \frac{\beta _j-2}{q_j}$, and $C < \infty$ is a constant that depends on $(q_j),(\beta _j)$.

For the sake of completeness we also state the one-dimensional version of Theorem 2.8.

### Corollary 2.12

Let *X* be a stochastic process on a bounded interval *I* such that

$$\begin{aligned} |X(x)-X(y)| \le \sum _{j=1}^J |A_j(x,y)| \end{aligned}$$

for all $x,y \in I$, where $A_j$, $j=1,\ldots ,J$, are measurable and satisfy

$$\begin{aligned} {\mathbb {E}}|A_j(x,y)|^{q_j} \le C' |x-y|^{\alpha _j} \end{aligned}$$

with $$q_j \ge 1$$, $$\alpha _j > 1$$, and $$C' < \infty $$.

Then *X* has a continuous modification ${\hat{X}}$ that satisfies, for any $\gamma < \min _j \frac{\alpha _j-1}{q_j}$,

$$\begin{aligned} |{\hat{X}}(x)-{\hat{X}}(y)| \le C_\gamma |x-y|^{\gamma } \end{aligned}$$

with a random variable $C_\gamma$ with ${\mathbb {E}}[C_\gamma ^{q_{\text {min}}}] < \infty$ where $q_{\text {min}} = \min _j q_j$.

### Further variations on the GRR theme

We give some additional results that are similar or come as consequence of Lemma 2.1. This demonstrates the flexibility and generality that our lemma provides. We do not aim for a complete survey of all implications of the lemma.

We begin by proving the result of Lemma 2.1 under slightly weaker assumptions. The assumptions may seem a bit at random, but they will turn out to be what we need in the proof of Theorem 4.1.

#### Lemma 2.13

Consider the same conditions as in Lemma 2.1, but instead of (5), we assume the following weaker condition. Let $r_j > 1$ and $\theta _j > 0$ such that $\frac{\beta _{1j}-2}{q_{1j}} < \theta _j$ for $j=1,\ldots ,J_1$.^1^ Suppose that for some small $c > 0$, e.g. $c \le |I_1|/4$, we have

14 $$\begin{aligned} \begin{aligned}&|G(x_1,x_2)-G(y_1,y_2)| \\&\quad \le \sum ^{J_1}_{j=1} \sum _{k=0}^{\lfloor \log _{r_j}(c/|x_1-y_1|) \rfloor } r_j^{-k\theta _j} |A_{1j}(z_1+r_j^k(x_1-z_1),z_1+r_j^k(y_1-z_1);x_2)| \\&\qquad +\, \sum ^{J_2}_{j=1} |A_{2j}(y_1;x_2,y_2)| \end{aligned} \end{aligned}$$

for $(x_1,x_2),(y_1,y_2) \in I_1 \times I_2$ and $z_1 \in I_1$ whenever $|x_1-z_1| \vee |y_1-z_1| \le 2|x_1-y_1|$ and all the points appearing in the sum are also in the domain $I_1$.

Then the result of Lemma 2.1 still holds, with the constant *C* depending also on $(r_j),(\theta _j)$.

#### Proof

We proceed similarly as in the proof of Lemma 2.1. We pick the sequence $I^n_i$ a bit more carefully. Let $d_i > 0$, $R_i > 1$, $i=1,2$, be as in the proof of Lemma 2.1, and recall that we can freely pick $R_i \ge 9$. It is not hard to see that we can then pick a sequence of rectangles $I^n_1 \times I^n_2$ in such a way that

- $$|I^n_i| = \frac{1}{9} R_i^{-n}d_i$$,
- $$\frac{1}{9} R_i^{-n}d_i \le {\text {dist}}(I^n_i,I^{n+1}_i) \le R_i^{-n}d_i$$,
- $${\text {dist}}(x_i,I^n_i) \rightarrow 0$$ as $$n \rightarrow \infty $$,

and another analogous sequence of rectangles for $(y_1,y_2)$ that begins with the same $I^0_1 \times I^0_2$.

The proof proceeds in the same way, but instead of the assumption (5), we apply (14) with some $z_1$ that we pick now.

Let $n \in {\mathbb {N}}$. We pick $z_1 \mathrel {\mathop :}=\inf (I^n_1 \cup I^{n-1}_1)$ if this point is in the left half of $I_1$, and $z_1 = \sup (I^n_1 \cup I^{n-1}_1)$ otherwise. From the defining properties of the sequence $(I^n_1)$ it follows that $|u_1-z_1| \vee |v_1-z_1| \le 2|u_1-v_1|$ for all $u_1 \in I^n_1$, $v_1 \in I^{n-1}_1$. Moreover, all the points $z_1+r^k(u_1-z_1)$ and $z_1+r^k(v_1-z_1)$, $k \le \lfloor \log _r(c/|x_1-y_1|) \rfloor$, are inside $I_1$ because $|r^k(u_1-z_1)| \le \frac{c}{|u_1-v_1|}|u_1-z_1| \le 2c$ and we have chosen $z_1$ to be more than distance $|I_1|/2 \ge 2c$ away (in the $u_1$ resp. $v_1$ direction) from the end of the interval $I_1$.

We now have to bound

$$\begin{aligned} \sum _k \fint _{I^n_1}\fint _{I^{n-1}_1}\fint _{I^n_2} r^{-k\theta _j} |A_{1j}(z_1+r^k(u_1-z_1),z_1+r^k(v_1-z_1);u_2)| \,du_2\,dv_1\,du_1 \end{aligned}$$

With the transformation $\phi _k(u_1) = z_1+r^k(u_1-z_1)$ we get

$$\begin{aligned}&\fint _{I^n_1}\fint _{I^{n-1}_1}\fint _{I^n_2} r^{-k\theta _j} |A_{1j}(z_1+r^k(u_1-z_1),z_1+r^k(v_1-z_1);u_2)| \\&\quad = r^{-k\theta _j} \fint _{\phi _k(I^n_1)}\fint _{\phi _k(I^{n-1}_1)}\fint _{I^n_2} |A_{1j}(u_1,v_1;u_2)| \\&\quad \le C r^{-k\theta _j} (r^k R_1^{-n} d_1)^{\beta _{1j}/q_{1j}} \fint _{\phi _k(I^n_1)}\fint _{\phi _k(I^{n-1}_1)}\fint _{I^n_2} \frac{|A_{1j}(u_1,v_1;u_2)|}{|u_1-v_1|^{\beta _{1j}/q_{1j}}}\\&\quad \le C r^{-k\theta _j} (r^k R_1^{-n} d_1)^{\beta _{1j}/q_{1j}} \left( \fint _{\phi _k(I^n_1)}\fint _{\phi _k(I^{n-1}_1)}\fint _{I^n_2} \frac{|A_{1j}(u_1,v_1;u_2)|^{q_{1j}}}{|u_1-v_1|^{\beta _{1j}}} \right) ^{1/q_{1j}} \\&\quad \le C r^{-k\theta _j} (r^k R_1^{-n} d_1)^{\beta _{1j}/q_{1j}} \left( (r^k R_1^{-n} d_1)^{-2} (R_2^{-n} d_2)^{-1} M_{1j} \right) ^{1/q_{1j}} \\&\quad = C r^{k((\beta _{1j}-2)/q_{1j}-\theta _j)} \left( (R_1^{-n} d_1)^{\beta _{1j}-2} (R_2^{-n} d_2)^{-1} M_{1j} \right) ^{1/q_{1j}}. \end{aligned}$$

Since we assumed $\frac{\beta _{1j}-2}{q_{1j}} < \theta _j$ this bound sums in *k* to

$$\begin{aligned} C \left( (R_1^{-n} d_1)^{\beta _{1j}-2} (R_2^{-n} d_2)^{-1} M_{1j} \right) ^{1/q_{1j}} \end{aligned}$$

which is the same bound as in the proof of Lemma 2.1. The rest of the proof is the same as in Lemma 2.1. $\square$

The following corollary is only used for Theorem 3.8.

#### Corollary 2.14

Consider the same conditions as in Lemma 2.1. For $x_1 \in I_1$, consider $G(x_1,\cdot )$ as an element in the space of continuous functions $C^0(I_2)$. Then the *p*-variation of $x_1 \mapsto G(x_1,\cdot )$ is at most

$$\begin{aligned} C \sum _j M_{1j}^{1/q_{1j}} |I_1|^{\gamma ^{(1)}_{1j}} + C \sum _j M_{2j}^{1/q_{2j}} |I_1|^{\gamma ^{(1)}_{2j}}, \end{aligned}$$

where $p = \max _{i,j} \frac{q_{ij}}{1+\gamma ^{(1)}_{ij} q_{ij}} = \max _j \frac{q_{1j}}{\beta _{1j}-1-b} \vee \max _j \frac{q_{2j}}{(\beta _{2j}-2)b}$ (with a choice of $b \ge 0$), and *C* does not depend on $|I_1|$.

#### Proof

Let $t^0< t^1< \cdots < t^n$ be a partition of $I_1$. The *p*-variation of $x_1 \mapsto G(x_1,\cdot ) \in C^0(I_2)$ is

$$\begin{aligned} \sup _{\text {partitions of }I_1} \left( \sum _k \sup _{x_2 \in I_2} |G(t^k,x_2)-G(t^{k-1},x_2)|^p \right) ^{1/p}. \end{aligned}$$

We estimate the differences using Lemma 2.1, applied to $[t^{k-1},t^k] \times I_2$. Observe that since consider the difference only in the first parameter of *G*, the constant *C* in the statement of Lemma 2.1 does not depend on the size of $[t^{k-1},t^k]$, as we explained in Remark 2.3. Hence we have

$$\begin{aligned}&|G(t^k,x_2)-G(t^{k-1},x_2)| \le C \sum _j \left( M_{1j} \big |_{[t^{k-1},t^k]} \right) ^{1/q_{1j}} |t^k-t^{k-1}|^{\gamma ^{(1)}_{1j}}\\&\quad +\, C \sum _j \left( M_{2j}\big |_{[t^{k-1},t^k]} \right) ^{1/q_{2j}} |t^k-t^{k-1}|^{\gamma ^{(1)}_{2j}} \end{aligned}$$

for all $x_2 \in I_2$, where we denote by $M_{1j}\big |_{[s,t]}$ and $M_{2j}\big |_{[s,t]}$ the integrals in (6) and (7) restricted to $[s,t] \times [s,t] \times I_2$ and $[s,t] \times I_2 \times I_2$, respectively.

Similarly to [6, Corollary A.3], we can show that

$$\begin{aligned} \omega (s,t) = C^p \sum _j \left( M_{1j}\big |_{[s,t]} \right) ^{p/q_{1j}} |s-t|^{p\gamma ^{(1)}_{1j}}+ C^p \sum _j \left( M_{2j}\big |_{[s,t]} \right) ^{p/q_{2j}} |s-t|^{p\gamma ^{(1)}_{2j}} \end{aligned}$$

is a control. $$\square $$

## Continuity of SLE in $\kappa$ and *t*

In this section we show the main results Theorems 1.1 and 1.2. We adopt notations and prerequisite from [10]. For the convenience of the reader, we quickly recall some important notations.

Let $U:[0,1]\rightarrow \mathbb {R}$ be continuous. The Loewner differential equation is the following initial value ODE

15 $$\begin{aligned} \partial _t g_t(z) = \frac{2}{g_t(z) -U(t)},\quad g_0(z) = z \in \mathbb {H}. \end{aligned}$$

For each $z\in \mathbb {H}$, the ODE has a unique solution up to a time $T_z=\sup \{t>0{:}\,|g_t(z)-U(t)|>0\} \in (0,\infty ]$. For $t \ge 0$, let $H_t = \{z\in \mathbb {H}{:}\,T_z>t\}$. It is known that $g_t$ is a conformal map from $H_t$ onto $\mathbb {H}.$ Define $f_t = g_t^{-1}$ and $\hat{f}_t = f_t( \cdot + U(t))$. One says that $\lambda$ generates a curve $\gamma$ if

16 $$\begin{aligned} \gamma (t):=\lim _{y\rightarrow 0^+} f_t(iy+U(t)) \end{aligned}$$

exists and is continuous in $t\in [0,1]$. This is equivalent to saying that there exists a continuous ${\overline{{\mathbb {H}}}}$-valued path $\gamma$ such that for each $t \in [0,1]$, the domain $H_t$ is the unbounded connected component of ${\mathbb {H}}{\setminus } \gamma [0,t]$.

It is known [16, 18] that for fixed $\kappa \in [0,\infty )$, the driving function $U=\sqrt{\kappa }B$, where *B* is a standard Brownian motion, almost surely generates a curve, which we will denote by $\gamma (\cdot ,\kappa )$ or $\gamma ^\kappa$. But we do not know whether given a Brownian motion *B*, almost surely all driving functions $\sqrt{\kappa }B$, $\kappa \ge 0$, simultaneously generate a curve. Furthermore, simulations suggest that for a fixed sample of *B*, the curve $\gamma ^\kappa$ changes continuously in $\kappa$, but only partial proofs have been found so far. We remark that this question is not trivial to answer because in general, the trace does not depend continuously on its driver, as [14, Example  4.49] shows.

In [10] the authors show that in the range $\kappa \in {[0, 8(2-\sqrt{3})[} \approx {[0, 2.1[}$, the answer to both of the above questions is positive. Our result Theorem 3.2 improves the range to $\kappa \in {[0, 8/3[}$.

We will often use the following bounds for the moments of $|{\hat{f}}_t'(iy)|$ that have been shown by Johansson Viklund and Lawler [9]. In order to state them, we use the following notation. Let $\kappa \ge 0$. Set

17 $$\begin{aligned} \begin{aligned} r_c = r_c(\kappa )&:= \frac{1}{2}+\frac{4}{\kappa },\\ \lambda (r) = \lambda (\kappa ,r)&:= r\left( 1+\frac{\kappa }{4}\right) -\frac{\kappa r^2}{8},\\ \zeta (r) = \zeta (\kappa ,r)&:= r-\frac{\kappa r^2}{8} \end{aligned} \end{aligned}$$

for $$r<r_c(\kappa )$$.

With the scaling invariance of SLE, [9, Lemma 4.1] implies the following.

### Lemma 3.1

[5, Lemma 2.1]^2^ Let $\kappa > 0$, $r < r_c(\kappa )$. There exists a constant $C<\infty$ depending only on $\kappa$ and *r* such that for all $t,y \in {]0,1]}$

$$\begin{aligned} {\mathbb {E}}[|{\hat{f}}_t'(iy)|^{\lambda (r)}] \le C a(t) y^{\zeta (r)} \end{aligned}$$

where $$a(t) = a(t,\zeta (r)) = t^{-\zeta (r)/2} \vee 1$$.

Moreover, *C* can be chosen independently of $\kappa$ and *r* when $\kappa$ is bounded away from 0 and $\infty$, and *r* is bounded away from $-\infty$ and $r_c(\kappa )$.^3^

Now, for a standard Brownian motion *B*, and an SLE$_\kappa$ flow driven by $\sqrt{\kappa }B$, we write ${\hat{f}}^\kappa _t$, $\gamma ^\kappa$, etc.

We also use the following notation from [9].

$$\begin{aligned} v(t,\kappa ,y) := \int _0^y |({\hat{f}}^\kappa _t)'(iu)| \, du. \end{aligned}$$

Observe that $v(t,\kappa ,\cdot )$ is decreasing in *y* and

$$\begin{aligned} |{\hat{f}}^\kappa _t(iy_1)-{\hat{f}}^\kappa _t(iy_2)| \le \int _{y_1}^{y_2} |({\hat{f}}^\kappa _t)'(iu)| \, du = |v(t,\kappa ,y_1)-v(t,\kappa ,y_2)|. \end{aligned}$$

Therefore $\lim _{y\searrow 0}{\hat{f}}^\kappa _t(iy)$ exists if $v(t,\kappa ,y)<\infty$ for some $y>0$. For fixed *t*, $\kappa$, this happens almost surely because Lemma 3.1 implies

$$\begin{aligned} {\mathbb {E}}v(t,\kappa ,y) = \int _0^y {\mathbb {E}}|({\hat{f}}^\kappa _t)'(iu)| \, du < \infty . \end{aligned}$$

So we can define

$$\begin{aligned} \gamma (t,\kappa ) = {\left\{ \begin{array}{ll} \lim _{y \searrow 0} {\hat{f}}^\kappa _t (iy) &{}\quad \text {if the limit exists,}\\ \infty &{}\quad \text {otherwise,} \end{array}\right. } \end{aligned}$$

as a random variable. Note that with this definition we can still estimate

$$\begin{aligned} |\gamma (t,\kappa )-{\hat{f}}^\kappa _t(iy)| \le v(t,\kappa ,y). \end{aligned}$$

### Almost sure regularity of SLE in $(t,\kappa )$

In this subsection, we prove our first main result.

#### Theorem 3.2

Let $0< \kappa _-< \kappa _+ < 8/3$. Let *B* be a standard Brownian motion. Then almost surely the SLE$_\kappa$ trace $\gamma ^\kappa$ driven by $\sqrt{\kappa }B$ exists for all $\kappa \in [\kappa _-,\kappa _+]$. Moreover, there exists a random variable *C*, depending on $\kappa _-$, $\kappa _+$, such that

$$\begin{aligned} |\gamma (t,\kappa )-\gamma (s,{\tilde{\kappa }})| \le C (|t-s|^\alpha + |\kappa -{\tilde{\kappa }}|^\eta ) \end{aligned}$$

for all $t,s \in [0,1]$, $\kappa ,{\tilde{\kappa }} \in [\kappa _-,\kappa _+]$ where $\alpha , \eta > 0$ depend on $\kappa _+$. Moreover, *C* can be chosen to have finite $\lambda$th moment for some $\lambda >1$.

The theorem should be still true near $\kappa \approx 0$ (Without any integrability statement for *C*, it is shown in [10].), but due to complications in applying Lemma 3.1 (cf. [10, Proof of Lemma 3.3]), we decided to omit it.

As in [5], we will estimate moments of the increments of $\gamma$, using Lemma 3.1. We need to be a little careful, though, when applying Lemma 3.1, that the exponents do depend on $\kappa$. Since we are going to apply that estimate a lot, let us agree on the following.

For every $\kappa > 0$, we will choose some $r_\kappa < r_c(\kappa )$, and we will call $\lambda _\kappa = \lambda (\kappa , r_\kappa )$ and $\zeta _\kappa = \zeta (\kappa , r_\kappa )$ [where $r_c$, $\lambda$, and $\zeta$ are defined in (17)]. (The exact choices of $r_\kappa$ will be decided later.)

We will use the following moment estimates.

#### Proposition 3.3

Let $0< \kappa _-< \kappa _+ < \infty$. Let $t,s \in [0,1]$, $\kappa , {\tilde{\kappa }} \in [\kappa _-, \kappa _+]$, and $p \in [1,1+\frac{8}{\kappa _+}[$. Then (with the above notation) if $\lambda _\kappa \ge 1$, then

$$\begin{aligned} {\mathbb {E}}|\gamma (t,\kappa )-\gamma (s,\kappa )|^{\lambda _\kappa }&\le C (a(t,\zeta _\kappa )+a(s,\zeta _\kappa )) \, | t-s|^{(\zeta _\kappa +\lambda _\kappa )/2},\\ {\mathbb {E}}|\gamma (s,\kappa )-\gamma (s,{\tilde{\kappa }}) |^p&\le C|\sqrt{\kappa }-\sqrt{{\tilde{\kappa }}}|^p, \end{aligned}$$

where $C<\infty$ depends on $\kappa _-$, $\kappa _+$, *p*, and the choice of $r_\kappa$ (see above).

#### Remark 3.4

Note that $|\sqrt{\kappa }-\sqrt{{\tilde{\kappa }}}| \le C|\kappa -{\tilde{\kappa }}|$ if $\kappa ,{\tilde{\kappa }}$ are bounded away from 0.

The first estimate is just [5, Lemma 3.2].

The second estimate follows from the following result (which we will prove in Sect. 5) and Fatou’s lemma.

#### Proposition 3.5

Let $0< \kappa _-< \kappa _+ < \infty$ and $\kappa ,{\tilde{\kappa }} \in [\kappa _-,\kappa _+]$. Let $t \in [0,T]$, $\delta \in {]0,1]}$, and $|x| \le \delta$. Then, for $1 \le p < 1+\frac{8}{\kappa _+}$, there exists $C < \infty$, depending on $\kappa _-$, $\kappa _+$, *T*, and *p*, such that

$$\begin{aligned} {\mathbb {E}}|{\hat{f}}^\kappa _t(x+i\delta )-{\hat{f}}^{{\tilde{\kappa }}}_t (x+i\delta )|^p \le C|\sqrt{\kappa }-\sqrt{{\tilde{\kappa }}}|^p. \end{aligned}$$

If $p > 1+\frac{8}{\kappa _+}$, then for any $\varepsilon > 0$ there exists $C < \infty$, depending on $\kappa _-$, $\kappa _+$, *T*, *p*, and $\varepsilon$, such that

$$\begin{aligned} {\mathbb {E}}|{\hat{f}}^\kappa _t(x+i\delta )-{\hat{f}}^{{\tilde{\kappa }}}_t (x+i\delta )|^p \le C|\sqrt{\kappa }-\sqrt{{\tilde{\kappa }}}|^p \delta ^{1+\frac{8}{\kappa _+}-p-\varepsilon }. \end{aligned}$$

#### Remark 3.6

Following the proof of [10], in particular using [10, Lemma 2.3] and Lemma 3.1, we can show

$$\begin{aligned} {\mathbb {E}}|{\hat{f}}^\kappa _t(x+i\delta )-{\hat{f}}^{{\tilde{\kappa }}}_t (x+i\delta )|^{2\lambda -\varepsilon } \le C |\sqrt{\kappa }-\sqrt{{\tilde{\kappa }}}|^{2\lambda -\varepsilon } \delta ^{-\lambda +\zeta -\varepsilon }. \end{aligned}$$

If we use this estimate instead, we can estimate

$$\begin{aligned} |\gamma (t,\kappa )-\gamma (s,{\tilde{\kappa }})|&\le |\gamma (t,\kappa )-\gamma (s,\kappa )| + | \gamma (s,\kappa )-\gamma (s,{\tilde{\kappa }})|\\&\le |\gamma (t,\kappa )-\gamma (s,\kappa )| \\&\quad +\, |\gamma (s,\kappa )-{\hat{f}}^\kappa _s(iy)| + |{\hat{f}}^\kappa _s(iy)-{\hat{f}}^{{\tilde{\kappa }}}_s(iy)| + |{\hat{f}}^{{\tilde{\kappa }}}_s(iy)-\gamma (s,{\tilde{\kappa }})| \end{aligned}$$

with $y = |\Delta \kappa |$. Then, with

$$\begin{aligned} {\mathbb {E}}|\gamma (t,\kappa )-\gamma (s,\kappa )|^\lambda&\le C |t-s|^{(\zeta +\lambda )/2},\\ {\mathbb {E}}|\gamma (s,\kappa )-{\hat{f}}^\kappa _s(iy)|^\lambda&\le Cy^{\zeta +\lambda } = C |\kappa -{\tilde{\kappa }}|^{\zeta +\lambda },\\ {\mathbb {E}}|{\hat{f}}^\kappa _s(iy)-{\hat{f}}^{{\tilde{\kappa }}}_s (iy)|^{2\lambda -\varepsilon }&\le C |\kappa -{\tilde{\kappa }}|^{\zeta +\lambda -\varepsilon }, \end{aligned}$$

Theorem 2.8 applies if $(\frac{\zeta +\lambda }{2})^{-1}+(\zeta +\lambda )^{-1} < 1 \iff \zeta +\lambda > 3$, which happens when $\kappa \in {[0,8(2-\sqrt{3})[} \cup {]8(2+\sqrt{3}),\infty [}$ and with an appropriate choice of *r*. Hence, we recover the continuity of SLE in the same range as in [10].

Notice that for fixed $\kappa > 0$ the maximal value that $\zeta +\lambda$ can attain is $\frac{\kappa }{4}\left( \frac{1}{2}+\frac{4}{\kappa }\right) ^2$ which is (for $\kappa < 8$) less than $p = 1+\frac{8}{\kappa }$ as in our Proposition 3.3. In other words, Proposition 3.3 is really an improvement to [10].

Below we write $x^+ = x\vee 0$ for $x \in {\mathbb {R}}$.

#### Corollary 3.7

Under the same conditions as in Proposition 3.5 we have

$$\begin{aligned} {\mathbb {E}}|({\hat{f}}^\kappa _t)'(i\delta )-({\hat{f}}^{{\tilde{\kappa }}}_t)' (i\delta )|^p \le C|\sqrt{\kappa }-\sqrt{{\tilde{\kappa }}}|^p \delta ^{-p-(p-1-\frac{8}{{\tilde{\kappa }}}+\varepsilon )^+} \end{aligned}$$

where $C<\infty$ depends on $\kappa _-$, $\kappa _+$, *T*, *p*, and $\varepsilon$.

#### Proof

For a holomorphic function $f{:}\, {\mathbb {H}}\rightarrow {\mathbb {H}}$, Cauchy Integral Formula tells us that

$$\begin{aligned} f'(i\delta ) = \frac{1}{i2\pi } \int _\alpha \frac{f(w)}{(w-i\delta )^2} \, dw \end{aligned}$$

where we let $\alpha$ be a circle of radius $\delta /2$ around $i\delta$. Consequently,

$$\begin{aligned} |({\hat{f}}^\kappa _t)'(i\delta )-({\hat{f}}^{{\tilde{\kappa }}}_t)'(i\delta )| \le \frac{1}{2\pi } \int _\alpha \frac{|{\hat{f}}^\kappa _t(w)-{\hat{f}}^{{\tilde{\kappa }}}_t(w)|}{\delta ^2/4} \, |dw|. \end{aligned}$$

For all *w* on the circle $\alpha$ we have $\mathfrak {I}w \in [\delta /2,3\delta /2]$ and $\mathfrak {R}w \in [-\delta /2,\delta /2]$. Therefore Proposition 3.5 implies

$$\begin{aligned} {\mathbb {E}}|{\hat{f}}^\kappa _t(w)-{\hat{f}}^{{\tilde{\kappa }}}_t(w)|^p \le C|\Delta \sqrt{\kappa }|^p \delta ^{-(p-1-\frac{8}{{\tilde{\kappa }}}+\varepsilon )^+}. \end{aligned}$$

By Minkowski’s inequality,

$$\begin{aligned} {\mathbb {E}}|({\hat{f}}^\kappa _t)'(i\delta )-({\hat{f}}^{{\tilde{\kappa }}}_t)' (i\delta )|^p \le \left( \frac{1}{2\pi } \int _\alpha \frac{({\mathbb {E}}|{\hat{f}}^\kappa _t(w)-{\hat{f}}^{{\tilde{\kappa }}}_t (w)|^p)^{1/p}}{\delta ^2/4} \, |dw| \right) ^p, \end{aligned}$$

and the result follows since the length of $\alpha$ is $\pi \delta$. $\square$

With Proposition 3.3, we can now apply Theorem 2.8 to construct a Hölder continuous version of the map $\gamma = \gamma (t,\kappa )$, whose Hölder constants have some finite moments.

There is just one detail we still have to take into consideration. In order to apply Theorem 2.8, we have to use one common exponent $\lambda$ on the entire range of $\kappa$ where we want to apply the GRR lemma. Of course, we can choose new values for $\lambda$ again when we consider a different range of $\kappa$.

Alternatively, we could formulate our GRR version to allow exponents to vary with the parameters. But this will not be necessary since we can break our desired interval for $\kappa$ into subintervals.

#### Proof of Theorem 3.2

Consider the joint SLE$_\kappa$ process in some range $\kappa \in [\kappa _-,\kappa _+]$. We can assume that the interval $[\kappa _-,\kappa _+]$ is so small that $\lambda (\kappa )$ and $\zeta (\kappa )$ are almost constant. Otherwise, break $[\kappa _-,\kappa _+]$ into small subintervals and consider each of them separately.

We perform the proof in three parts. First we construct a continuous version ${\tilde{\gamma }}$ of $\gamma$ using Theorem 2.8. Then, using Lemma 2.1, we show that ${\tilde{\gamma }}$ is jointly Hölder continuous in both variables. Finally, we show that for each $\kappa$, the path ${\tilde{\gamma }}(\cdot ,\kappa )$ is indeed the SLE$_\kappa$ trace generated by $\sqrt{\kappa }B$.

**Part 1** For the first part, we would like to apply Theorem 2.8. There is just one technical detail we need to account for. In the estimates of Proposition 3.3, there is a singularity at time $t=0$, but we have not formulated Theorem 2.8 to allow $C'$ to have a singularity. Therefore, it is easier to apply Theorem 2.8 on the domain $[\varepsilon ,1] \times [\kappa _-,\kappa _+]$ with $\varepsilon > 0$. With $\varepsilon \searrow 0$, we obtain a continuous version of $\gamma$ on the domain $]0,1] \times [\kappa _-,\kappa _+]$. Due to the local growth property of Loewner chains, we must have $\lim _{t \searrow 0} \gamma (t,\kappa ) = 0$ uniformly in $\kappa$, so we actually have a continuous version of $\gamma$ on $[0,1] \times [\kappa _-,\kappa _+]$.^4^

Now we apply Proposition 3.3 on the domain $[\varepsilon ,1] \times [\kappa _-,\kappa _+]$. For this, we pick $\lambda \ge 1$, $r_\kappa < r_c(\kappa )$, and $p \in {[1,1+\frac{8}{\kappa _+}[}$ in such a way that $\lambda _\kappa = \lambda$ for all $\kappa \in [\kappa _-,\kappa _+]$. The condition to apply Theorem 2.8 is then $(\frac{\zeta +\lambda }{2})^{-1}+p^{-1} < 1$.

A computation shows that $\zeta +\lambda = \frac{\kappa }{4}r\left( 1+\frac{8}{\kappa }-r\right)$ attains its maximal value $\frac{\kappa }{4}\left( \frac{1}{2}+\frac{4}{\kappa }\right) ^2$ at $r = \frac{1}{2}+\frac{4}{\kappa } = r_c$. Note also that $\lambda (r_c) = 1+\frac{2}{\kappa }+\frac{3}{32}\kappa > 1$. Recall from above that we can pick any $p < 1+\frac{8}{\kappa }$. Therefore, the condition for the exponents is

$$\begin{aligned} \frac{2}{\frac{\kappa }{4}\left( \frac{1}{2}+ \frac{4}{\kappa }\right) ^2}+\frac{1}{1+\frac{8}{\kappa }}< 1 \iff \kappa < \frac{8}{3}. \end{aligned}$$

This completes the first part of the proof and gives us a continuous random field ${\tilde{\gamma }}$.

**Part 2** Now that we have a random continuous function ${\tilde{\gamma }}$, we can apply Lemma 2.1. As in the proof of Theorem 2.8, we show that the integrals (6) and (7) have finite expectation, and therefore are almost surely finite. Denoting $|A_1(t,s;\kappa )| \mathrel {\mathop :}=|\gamma (t,\kappa )-\gamma (s,\kappa )|$, $|A_2(s;\kappa ,{\tilde{\kappa }})| \mathrel {\mathop :}=|\gamma (s,\kappa )-\gamma (s,{\tilde{\kappa }})|$, and the corresponding integrals by $M_1, M_2$, we have by Proposition 3.3

$$\begin{aligned} {\mathbb {E}}M_1&\lesssim \iiint (a(t)+a(s)) |t-s|^{(\zeta +\lambda )/2 -\beta _1} \,dt\,ds\,d\kappa , \\ {\mathbb {E}}M_2&\lesssim \iiint |\kappa -{\tilde{\kappa }}|^{p-\beta _2} \,ds\,d\kappa \,d{\tilde{\kappa }}. \end{aligned}$$

Picking $\beta _1 = \frac{\zeta +\lambda }{2}+1-\varepsilon$, $\beta _2 = p+1-\varepsilon$, the condition for the exponents is again $(\frac{\zeta +\lambda }{2})^{-1}+p^{-1} < 1$. Additionally, we need to account for the singularity at $t=0$ in the first integrand. This is not a problem if the function $a(t) = t^{-\zeta /2} \vee 1$ is integrable.

To make $a(t) = t^{-\zeta /2} \vee 1$ integrable, we would like to have $\zeta < 2$.^5^ Recall that $\zeta = r-\frac{\kappa r^2}{8}$ from (17). In case $\kappa > 1$, we always have $\zeta < 2$. In case $\kappa \le 1$, we have $\zeta < 2$ for $r < \frac{4}{\kappa }(1-\sqrt{1-\kappa })$, or equivalently $\lambda (r) < 3-\sqrt{1-\kappa }$. Therefore we can certainly find *r* such that $\zeta < 2$ and $\zeta +\lambda \approx 2+(3-\sqrt{1-\kappa })$, and $p \approx 9 < 1+\frac{8}{\kappa }$. The condition $(\frac{\zeta +\lambda }{2})^{-1}+p^{-1} < 1$ is still fulfilled.

This proves the statements about the Hölder continuity of ${\tilde{\gamma }}$.

**Part 3** In the final part, we show that for each $\kappa$, the path ${\tilde{\gamma }}(\cdot ,\kappa )$ is indeed the SLE$_\kappa$ trace generated by $\sqrt{\kappa }B$.

First, we fix a countable dense subset $\mathcal {K}$ in $[\kappa _-,\kappa _+]$. There exists a set $\Omega _1$ of probability 1 such that for all $\omega \in \Omega _1$, all $\kappa \in \mathcal {K}$, $\gamma (\kappa ,t)$ exists and is continuous in *t*.

Since ${\tilde{\gamma }}$ is a version of $\gamma$, for all *t*,

$$\begin{aligned} {\mathbb {P}}\big ( \gamma (t,\kappa )= {\tilde{\gamma }}(t,\kappa ) \text { for all } \kappa \in \mathcal {K}\big )=1. \end{aligned}$$

Hence, there exists a set $\Omega _2$ with probability 1 such that for all $\omega \in \Omega _2$, we have $\gamma (t,\kappa )={\tilde{\gamma }}(t,\kappa )$ for all $\kappa \in \mathcal {K}$ and almost all *t*. Restricted to $\omega \in \Omega _3=\Omega _1\cap \Omega _2$, the previous statement is true for all $\kappa \in \mathcal {K}$ and all *t*. We claim that on the set $\Omega _3$ of probability 1, the path $t \mapsto {\tilde{\gamma }}(t,\kappa )$ is indeed the SLE$_\kappa$ trace driven by $\sqrt{\kappa }B$. This can be shown in the same way as [16, Theorem 4.7].

Indeed, fix $t \in [0,1]$ and let $H_t = f^\kappa _t({\mathbb {H}})$. We show that $H_t$ is the unbounded connected component of ${\mathbb {H}}{\setminus } {\tilde{\gamma }}([0,t],\kappa )$.^6^ Find a sequence of $\kappa _n \in \mathcal {K}$ with $\kappa _n \rightarrow \kappa$ and let $(f^{\kappa _n}_t)$ be the corresponding inverse Loewner maps. Since $\sqrt{\kappa _n}B \rightarrow \sqrt{\kappa }B$, the Loewner differential equation implies that $f^{\kappa _n}_t \rightarrow f^\kappa _t$ uniformly on each compact set of ${\mathbb {H}}$. By the chordal version of the Carathéodory kernel theorem (see [17, Theorem 1.8]) which can be easily shown with the obvious adaptions, it follows that $H^{\kappa _n}_t \rightarrow H_t$ in the sense of kernel convergence. Since $\kappa _n\in \mathcal {K}$, we have $H^{\kappa _n}_t = {\mathbb {H}}{\setminus } \gamma ([0,t],\kappa _n) = {\mathbb {H}}{\setminus } {\tilde{\gamma }}([0,t],\kappa _n)$. Therefore, the definitions of kernel convergence and the uniform continuity of ${\tilde{\gamma }}$ imply that $H_t$ is the unbounded connected component of ${\mathbb {H}}{\setminus } {\tilde{\gamma }}([0,t],\kappa )$. $\square$

By Theorem 3.2, we now know that with probability one, the SLE$_\kappa$ trace $\gamma = \gamma (t,\kappa )$ is jointly continuous in $[0,1] \times [\kappa _-,\kappa _+]$. Similarly, applying Corollary 2.14, we can show the following.

#### Theorem 3.8

Let $0< \kappa _-< \kappa _+ < 8/3$. Let $\gamma ^\kappa$ be the SLE$_\kappa$ trace driven by $\sqrt{\kappa }B$, and assume it is jointly continuous in $(t,\kappa ) \in [0,1] \times [\kappa _-,\kappa _+]$. Consider $\gamma ^\kappa$ as an element of $C^0([0,1])$ (with the metric $\Vert \cdot \Vert _\infty )$.

Then for some $0< p <1/\eta$ (with $\eta$ from Theorem 3.2), the *p*-variation of $\kappa \mapsto \gamma ^\kappa$, $\kappa \in [\kappa _-,\kappa _+]$, is a.s. finite and bounded by some random variable *C*, depending on $\kappa _-$, $\kappa _+$, that has finite $\lambda$th moment for some $\lambda >1$.

We know that for fixed $\kappa \le 4$, the SLE$_\kappa$ trace is almost surely simple. It is natural to expect that there is a common set of probability 1 where all SLE$_\kappa$ traces, $\kappa < 8/3$, are simple. This is indeed true.

#### Theorem 3.9

Let *B* be a standard Brownian motion. We have with probability 1 that for all $\kappa < 8/3$ the SLE$_\kappa$ trace driven by $\sqrt{\kappa }B$ is simple.

#### Proof

As shown in [18, Theorem 6.1], due to the independent stationary increments of Brownian motion, this is equivalent to saying that $K^\kappa _t \cap {\mathbb {R}}= \{0\}$ for all *t* and $\kappa$, where $K^\kappa _t = \{ z \in {\overline{{\mathbb {H}}}}\mid T^\kappa _z \le t \}$ (the upper index denotes the dependence on $\kappa$).

Let $(g_t(x))_{t \ge 0}$ satisfy (15) with $g_0(x)=x$ and driving function $U(t)=\sqrt{\kappa }B_t$. Then $X_t = \frac{g_t(x)-\sqrt{\kappa }B_t}{\sqrt{\kappa }}$ satisfies

$$\begin{aligned} dX_t = \frac{2/\kappa }{X_t}\, dt - dB_t, \end{aligned}$$

i.e. *X* is a Bessel process of dimension $1+\frac{4}{\kappa }$. The statement $K^\kappa _t \cap {\mathbb {R}}= \{0\}$ is equivalent to saying that $X_s \ne 0$ for all $x \ne 0$ and $s \in [0,t]$. This is a well-known property of Bessel processes, and stated in the lemma below. $\square$

#### Lemma 3.10

Let *B* be a standard Brownian motion and suppose that we have a family of stochastic processes $X^{\kappa ,x}$, $\kappa ,x>0$, that satisfy

$$\begin{aligned} X^{\kappa ,x}_t = x + B_t + \int _0^t \frac{2/\kappa }{X^{\kappa ,x}_s} \, ds, \quad t \in [0,T_{\kappa ,x}] \end{aligned}$$

where $$T_{\kappa ,x} = \inf \{ t \ge 0 \mid X^{\kappa ,x}_t=0 \}$$.

Then we have with probability 1 that $T_{\kappa ,x}=\infty$ for all $\kappa \le 4$ and $x>0$.

#### Proof

For fixed $\kappa \le 4$, see e.g. [14, Proposition 1.21]. To get the result simultaneously for all $\kappa$, use the property that if $\kappa < {\tilde{\kappa }}$ and $x>0$, then $X^{\kappa ,x}_t > X^{{\tilde{\kappa }},x}_t$ for all $t>0$, which follows from Grönwall’s inequality. $\square$

### Stochastic continuity of SLE$_\kappa$ in $\kappa$

In the previous section, we have shown almost sure continuity of SLE$_\kappa$ in $\kappa$ (in the range $\kappa \in [0,8/3[$). Weaker forms of continuity are easier to prove, and hold on a larger range of $\kappa$. We will show here that stochastic continuity (also continuity in $L^q({\mathbb {P}})$ sense for some $q>1$ depending on $\kappa$) for all $\kappa \ne 8$ is an immediate consequence of our estimates. Below we write $\Vert f \Vert _{C^\alpha [a,b]} := \sup \frac{|f(t)-f(s)|}{|t-s|^\alpha }$, with $\sup$ taken over all $s<t$ in [*a*, *b*].

#### Theorem 3.11

Let $\kappa > 0$, $\kappa \ne 8$. Then there exists $\alpha > 0$, $q > 1$, $r > 0$, and $C < \infty$ (depending on $\kappa )$ such that if ${\tilde{\kappa }}$ is sufficiently close to $\kappa$ (where “sufficiently close” depends on $\kappa )$, then

$$\begin{aligned} {\mathbb {E}}\left[ \Vert \gamma (\cdot , \kappa )-\gamma (\cdot , {\tilde{\kappa }}) \Vert _{C^\alpha [0,1]}^q \right] \le C |\kappa - {\tilde{\kappa }}|^r. \end{aligned}$$

In particular, if $\kappa _n \rightarrow \kappa$ exponentially fast, then $\Vert \gamma (\cdot , \kappa )-\gamma (\cdot , \kappa _n) \Vert _{C^\alpha [0,1]} \rightarrow 0$ almost surely.

Note that without sufficiently fast convergence of $\kappa _n \rightarrow \kappa$ it is not clear whether we can pass from $L^q$-convergence to almost sure convergence.

#### Proof

Fix $\kappa , {\tilde{\kappa }} \ne 8$. We apply Corollary 2.11 to the function $G: [0,1] \rightarrow {\mathbb {C}}$, $G(t) = \gamma (t,\kappa )-\gamma (t,{\tilde{\kappa }})$. We have

$$\begin{aligned} |G(t)-G(s)|&\le (|\gamma (t,\kappa )-\gamma (s,\kappa )|+|\gamma (t,{\tilde{\kappa }}) -\gamma (s,{\tilde{\kappa }})|) \, 1_{|t-s| \le |\kappa -{\tilde{\kappa }}|} \\&\qquad +\, (|\gamma (t,\kappa )-\gamma (t,{\tilde{\kappa }})|+| \gamma (s,\kappa )-\gamma (s,{\tilde{\kappa }})|) \, 1_{|t-s| > | \kappa -{\tilde{\kappa }}|}\\&=: A_1(t,s) + A_2(t,s) \end{aligned}$$

where by Proposition 3.3

$$\begin{aligned} {\mathbb {E}}|A_1(t,s)|^\lambda&\le C (a^1(t)+a^1(s)) \, |t-s|^{(\zeta +\lambda )/2} \, 1_{|t-s| \le |\kappa -{\tilde{\kappa }}|},\\ {\mathbb {E}}|A_2(t,s)|^p&\le C|\kappa -{\tilde{\kappa }}|^p \, 1_{|t-s| > |\kappa -{\tilde{\kappa }}|}, \end{aligned}$$

for suitable $$\lambda \ge 1$$, $$p \in [1,1+\frac{8}{\kappa }[$$.

It follows that, for $\beta _1,\beta _2 > 0$,

$$\begin{aligned} {\mathbb {E}}\iint \frac{|A_1(t,s)|^\lambda }{|t-s|^{\beta _1}} \, dt \, ds&\le C \iint _{|t-s| \le |\kappa -{\tilde{\kappa }}|} (a^1(t)+a^1(s)) \, |t-s|^{(\zeta +\lambda )/2-\beta _1} \, dt \, ds \\&\le C |\kappa -{\tilde{\kappa }}|^{(\zeta +\lambda )/2-\beta _1+1},\\ {\mathbb {E}}\iint \frac{|A_2(t,s)|^p}{|t-s|^{\beta _2}} \, dt \, ds&\le C|\kappa -{\tilde{\kappa }}|^p \iint _{|t-s| > |\kappa -{\tilde{\kappa }}|} |t-s|^{-\beta _2} \, dt \, ds \\&\le C|\kappa -{\tilde{\kappa }}|^{p-\beta _2+1} \end{aligned}$$

if $$\zeta < 2$$ and $$\beta _1 < \frac{\zeta +\lambda }{2}+1$$.

Recall that if $\kappa \ne 8$ and ${\tilde{\kappa }}$ is sufficiently close to $\kappa$, then the parameters $\lambda ,\zeta$ are almost the same for $\kappa$ and ${\tilde{\kappa }}$, and (see the proof of Theorem 3.2) they can be picked such that $\zeta < 2$ and $\zeta +\lambda > 2$. Hence, we can pick $\beta _1,\beta _2 > 2$ such that $2< \beta _1 < \frac{\zeta +\lambda }{2}+1$ and $2< \beta _2< 1+p < 2+\frac{8}{\kappa }$.

The result follows from Corollary 2.11, where we take $\alpha = \frac{\beta _1-2}{\lambda } \wedge \frac{\beta _2-2}{p}$ and $q = \lambda \wedge p$, which implies

$$\begin{aligned} {\mathbb {E}}\left[ \Vert G \Vert _{C^\alpha [0,1]}^q \right] \le C {\mathbb {E}}\left[ \left( \iint \frac{|A_1(t,s)|^\lambda }{|t-s|^{\beta _1}} \, dt \, ds \right) ^{q/\lambda } + \left( \iint \frac{|A_2(t,s)|^p}{|t-s|^{\beta _2}} \, dt \, ds \right) ^{q/p} \right] . \end{aligned}$$

$$\square $$

#### Corollary 3.12

For any $\kappa > 0$, $\kappa \ne 8$ and any sequence $\kappa _n \rightarrow \kappa$ we then have $\Vert \gamma ^\kappa -\gamma ^{\kappa _n}\Vert _{p\text {-var};[0,1]} \rightarrow 0$ in probability, for any $p > (1 + \kappa / 8) \wedge 2$.

#### Proof

Theorem 3.11 immediately implies the statement with $\Vert \cdot \Vert _\infty$. To upgrade the result to Hölder and *p*-variation topologies, recall the following general fact which follows from the interpolation inequalities for Hölder and *p*-variation constants (see e.g. [6, Proposition 5.5]):

Suppose $X_n$, *X* are continuous stochastic processes such that for every $\varepsilon > 0$ there exists $M>0$ such that ${\mathbb {P}}(\Vert X_n\Vert _{p\text {-var};[0,T]} > M) < \varepsilon$ for all *n*. If $X_n \rightarrow X$ in probability with respect to the $\Vert \cdot \Vert _\infty$ topology, then also with respect to the $p'$-variation topology for any $p' > p$. The analogous statement holds for Hölder topologies with $\alpha ' < \alpha \le 1$.

In order to apply this fact, we can use [5, Theorem 5.2 and 6.1] which bound the moments of $\Vert \gamma \Vert _{p\text {-var}}$ and $\Vert \gamma \Vert _{C^\alpha }$. The values for *p* and $\alpha$ have also been computed there. $\square$

## Convergence results

Here we prove a stronger version of Theorem 3.2, namely uniform convergence (even convergence in Hölder sense) of ${\hat{f}}^\kappa _t(iy)$ as $y \searrow 0$. For this result, we really use the full power of Lemma 2.1 (actually Lemma 2.13 as we will explain later). We point out that this is a stronger result than Theorem 1.1, and that our previous proofs of Theorem 1.1 and 1.2 do not rely on this section.

The Hölder continuity in Theorem 3.2 induces an (inhomogeneous) Hölder space, with (inhomogeneous) Hölder constant that we denote by

$$\begin{aligned} \Vert \gamma \Vert _{C^{\alpha ,\eta }} \mathrel {\mathop :}=\sup _{(t,\kappa ) \ne (s,{\tilde{\kappa }})} \frac{|\gamma (t,\kappa )-\gamma (s,{\tilde{\kappa }})|}{|t-s|^\alpha +|\kappa -{\tilde{\kappa }}|^\eta }. \end{aligned}$$

As before, we write

$$\begin{aligned} v(t,\kappa ,y) = \int _0^y |({\hat{f}}^\kappa _t)'(iu)| \, du. \end{aligned}$$

### Theorem 4.1

Let $\kappa _- > 0$, $\kappa _+ < 8/3$. Then $\Vert v(\cdot ,\cdot ,y)\Vert _{\infty ; [0,1] \times [\kappa _-,\kappa _+]} \searrow 0$ almost surely as $y \searrow 0$. In particular, ${\hat{f}}^\kappa _t(iy)$ converges uniformly in $(t,\kappa ) \in [0,1] \times [\kappa _-,\kappa _+]$ as $y \searrow 0$.

Moreover, both functions converge also almost surely in the same Hölder space $C^{\alpha ,\eta }([0,1] \times [\kappa _-,\kappa _+])$ as in Theorem 3.2.

Moreover, the (random) Hölder constants of $v(\cdot ,\cdot ,y)$ and $(t,\kappa ) \mapsto |\gamma (t,\kappa )-{\hat{f}}^\kappa _t(iy)|$ satisfy

$$\begin{aligned} {\mathbb {E}}[\Vert v(\cdot ,\cdot ,y)\Vert _{C^{\alpha ,\eta }}^\lambda ] \le Cy^r \quad \text {and}\quad {\mathbb {E}}[\Vert \gamma (\cdot ,\cdot ) - {\hat{f}}^\cdot _\cdot (iy)\Vert _{C^{\alpha ,\eta }}^\lambda ] \le Cy^r \end{aligned}$$

for some $\lambda > 1$, $r>0$ and $C<\infty$, and all $y \in {]0,1]}$.

As a consequence, we obtain also an improved version of [10, Lemma 3.3].

### Corollary 4.2

Let $\kappa _- > 0$, $\kappa _+ < 8/3$. Then there exist $\beta < 1$ and a random variable $c(\omega ) < \infty$ such that almost surely

$$\begin{aligned} \sup _{(t,\kappa ) \in [0,1] \times [\kappa _-,\kappa _+]} |({\hat{f}}^\kappa _t)'(iy)| \le c(\omega ) y^{-\beta } \end{aligned}$$

for all $$y \in {]0,1]}$$.

### Proof

By Koebe’s 1/4-Theorem we have $y|({\hat{f}}^\kappa _t)'(iy)| \le 4{\text {dist}}({\hat{f}}^\kappa _t(iy), \partial H^\kappa _t) \le 4v(t,\kappa ,y)$. Theorem 4.1 and the Borel–Cantelli lemma imply

$$\begin{aligned} \Vert v(\cdot ,\cdot ,2^{-n})\Vert _\infty \le 2^{-nr'} \end{aligned}$$

for some $r'>0$ and sufficiently large (depending on $\omega$) *n*. The result then follows by Koebe’s distortion theorem (with $\beta = 1-r'$). $\square$

The same method as Theorem 4.1 can be used to show the existence and Hölder continuity of the SLE$_\kappa$ trace for fixed $\kappa \ne 8$, avoiding a Borel-Cantelli argument. The best way of formulating this result is the terminology in [5].

For $\delta \in {]0,1[}$, $q \in {]1,\infty [}$, define the fractional Sobolev (Slobodeckij) semi-norm of a measurable function $x{:}\,[0,1] \rightarrow {\mathbb {C}}$ as

$$\begin{aligned} \Vert x\Vert _{W^{\delta ,q}} := \left( \int _0^1 \int _0^1 \frac{|x(t)-x(s)|^q}{|t-s|^{1+\delta q}} \, ds \, dt \right) ^{1/q}. \end{aligned}$$

As a consequence of the (classical) one-dimensional GRR inequality (see [6, Corollary A.2 and A.3]), we have that for all $\delta \in {]0,1[}$, $q \in {]1,\infty [}$ with $\delta -1/q > 0$, there exists a constant $C<\infty$ such that for all $x \in C[0,1]$ we have

$$\begin{aligned} \Vert x\Vert _{C^\alpha [s,t]} \le C \Vert x\Vert _{W^{\delta ,q}[s,t]} \end{aligned}$$

and

$$\begin{aligned} \Vert x\Vert _{p\text {-var};[s,t]} \le C |t-s|^\alpha \Vert x\Vert _{W^{\delta ,q}[s,t]}, \end{aligned}$$

where $p=1/\delta$ and $\alpha =\delta -1/q$, and $\Vert x\Vert _{C^\alpha [s,t]}$ and $\Vert x\Vert _{p\text {-var};[s,t]}$ denote the Hölder and *p*-variation constants of *x*, restricted to [*s*, *t*].

Fix $\kappa \ge 0$, and as before, let

$$\begin{aligned} v(t,y) = \int _0^y |{\hat{f}}_t'(iu)| \, du. \end{aligned}$$

Recall the notation (17), and let $\lambda =\lambda (r)$, $\zeta =\zeta (r)$ with some $r < r_c(\kappa )$.

The following result is proved similarly to Theorem 4.1.

### Theorem 4.3

Let $\kappa \ne 8$. Then for some $\alpha > 0$ and some $p < 1/\alpha$ there almost surely exists a continuous $\gamma {:}\, [0,1] \rightarrow {\overline{{\mathbb {H}}}}$ such that the function $t \mapsto {\hat{f}}_t(iy)$ converges in $C^\alpha$ and *p*-variation to $\gamma$ as $y \searrow 0$.

More precisely, let $\kappa \ge 0$ be arbitrary, $\zeta <2$ and $\delta \in {\left]0, \frac{\lambda +\zeta }{2\lambda } \right[}$. Then there exists a random measurable function $\gamma {:}\,[0,1] \rightarrow {\overline{{\mathbb {H}}}}$ such that

$$\begin{aligned} {\mathbb {E}}\Vert v(\cdot ,y)\Vert _{W^{\delta ,\lambda }}^{\lambda } \le C y^{\lambda +\zeta -2\delta \lambda } \quad \text {and} \quad {\mathbb {E}}\Vert \gamma -{\hat{f}}_\cdot (iy)\Vert _{W^{\delta ,\lambda }}^{\lambda } \le C y^{\lambda +\zeta -2\delta \lambda } \end{aligned}$$

for all $y \in {]0,1]}$, where *C* is a constant that depends on $\kappa$, *r*, and $\delta$. Moreover, a.s. $\Vert v(\cdot ,y)\Vert _{W^{\delta ,\lambda }} \rightarrow 0$ and $\Vert \gamma -{\hat{f}}_\cdot (iy)\Vert _{W^{\delta ,\lambda }} \rightarrow 0$ as $y \searrow 0$.

If additionally $\delta \in {\left]\frac{1}{\lambda }, \frac{\lambda +\zeta }{2\lambda } \right[}$, then the same is true for $\Vert \cdot \Vert _{1/\delta \text {-var}}$ and $\Vert \cdot \Vert _{C^\alpha }$ where $\alpha =\delta -1/\lambda$.

### Remark 4.4

The conditions for the exponents are the same as in [5]. In particular, the result applies to the (for SLE$_\kappa$) optimal *p*-variation and Hölder exponents.

### Proof of Theorem 4.1

We use the same setting as in the proof of Theorem 3.2. For $\kappa \le \kappa _+ < 8/3$, we choose $p \in [1, 1+\frac{8}{\kappa _+}[$, $r_\kappa < r_c(\kappa )$, $\lambda (\kappa ,r_\kappa ) = \lambda \ge 1$, and the corresponding $\zeta _\kappa = \zeta (\kappa ,r_\kappa )$ as in the proof of Theorem 3.2. Again, we assume that the interval $[\kappa _-,\kappa _+]$ is small enough so that $\lambda (\kappa )$ and $\zeta (\kappa )$ are almost constant.

**Step 1** We would like to show that *v* and ${\hat{f}}$ (defined above) are Cauchy sequences in the aforementioned Hölder space as $y \searrow 0$. Therefore we will take differences $|v(\cdot ,\cdot ,y_1)-v(\cdot ,\cdot ,y_2)|$ and $|{\hat{f}}(iy_1)-{\hat{f}}(iy_2)|$, and estimate their Hölder norms with our GRR lemma. Note that it is not a priori clear that $v(t,\kappa ,y)$ is continuous in $(t,\kappa )$, but $|v(t,\kappa ,y_1)-v(t,\kappa ,y_2)| = \int _{y_1}^{y_2} |({\hat{f}}^\kappa _t)'(iu)| \, du$ certainly is, so the GRR lemma can be applied to this function.

Consider the function

$$\begin{aligned} G(t,\kappa ) := v(t,\kappa ,y)-v(t,\kappa ,y_1) = \int _{y_1}^y |({\hat{f}}^\kappa _t)'(iu)| \, du. \end{aligned}$$

The strategy will be to show that the condition of Lemma 2.1 is satisfied almost surely for *G*. As in the proof of Kolmogorov’s continuity theorem, we do this by showing that the expectation of the integrals (6), (7) are finite (after defining suitable $A_{1j}$, $A_{2j}$) and converge to 0 as $y \searrow 0$. In particular, they are almost surely finite, so Lemma 2.1 then implies that *G* is Hölder continuous, with Hölder constant bounded in terms of the integrals (6), (7).

We would like to infer that almost surely the functions $v(\cdot ,\cdot ,y)$, $y>0$, form a Cauchy sequence in the Hölder space $C^{\alpha ,\eta }$. But this is not immediately clear, therefore we will bound the integrals (6), (7) by expressions that are decreasing in *y*. We will also define $A_{1j}$, $A_{2j}$ here.

In order to do so, we estimate

$$\begin{aligned}&|G(t,\kappa )-G(s,{\tilde{\kappa }})| \\&\quad \le \int _0^y \left| |({\hat{f}}^\kappa _t)'(iu)|-|({\hat{f}}^\kappa _s)'(iu)| \right| \, du + \int _0^y \left| |({\hat{f}}^\kappa _s)'(iu)|-|({\hat{f}}^{{\tilde{\kappa }}}_s)'(iu)| \right| \, du\\&\quad \le \int _0^y |({\hat{f}}^\kappa _t)'(iu)-({\hat{f}}^\kappa _s)'(iu)| \, du + \int _0^y |({\hat{f}}^\kappa _s)'(iu)-({\hat{f}}^{{\tilde{\kappa }}}_s)'(iu)| \, du\\&\quad =: A_{1*}(t,s;\kappa )+A_{2*}(s;\kappa ,{\tilde{\kappa }}), \end{aligned}$$

Moreover, the function ${\hat{G}}(t,\kappa ) := {\hat{f}}^\kappa _t(iy)-{\hat{f}}^\kappa _t(iy_1)$ also satisfies

$$\begin{aligned} |{\hat{G}}(t,\kappa )-{\hat{G}}(s,{\tilde{\kappa }})| \le A_{1*}(t,s;\kappa )+A_{2*}(s;\kappa ,{\tilde{\kappa }}). \end{aligned}$$

Therefore all our considerations for *G* apply also to ${\hat{G}}$.

We want to estimate the difference $|({\hat{f}}^\kappa _s)'(iu)-({\hat{f}}^{{\tilde{\kappa }}}_s)'(iu)|$ differently for small and large *u* (relatively to $|\Delta \kappa |$), therefore we split $A_{2*}$ into

$$\begin{aligned} A_{2*}(s;\kappa ,{\tilde{\kappa }})&= \int _0^{y \wedge |\kappa -{\tilde{\kappa }}|^{p/(\zeta +\lambda )}} |({\hat{f}}^\kappa _s)'(iu)-({\hat{f}}^{{\tilde{\kappa }}}_s)'(iu)| \, du \\&\qquad +\, \int _{y \wedge |\kappa -{\tilde{\kappa }}|^{p/(\zeta +\lambda )}}^y |({\hat{f}}^\kappa _s)'(iu)-({\hat{f}}^{{\tilde{\kappa }}}_s)'(iu)| \, du\\&=: A_{21}(s;\kappa ,{\tilde{\kappa }})\\&\qquad +\,A_{22}(s;\kappa ,{\tilde{\kappa }}). \end{aligned}$$

We would like to apply Lemma 2.1 with these choices of $A_{1*}, A_{21}, A_{22}$. We denote the integrals (6), (7) by

$$\begin{aligned} M_{1*}&\mathrel {\mathop :}=&\iiint \frac{|A_{1*}(t,s;\kappa )|^\lambda }{|t-s|^{\beta _1}} \, ds \, dt \, d\kappa ,\\ M_{21}&\mathrel {\mathop :}=&\iiint \frac{|A_{21}(s;\kappa ,{\tilde{\kappa }})|^\lambda }{|\kappa -{\tilde{\kappa }}|^{\beta _2}} \, ds \, d\kappa \, d{\tilde{\kappa }},\\ M_{22}&\mathrel {\mathop :}=&\iiint \frac{|A_{22}(s;\kappa ,{\tilde{\kappa }})|^p}{|\kappa -{\tilde{\kappa }}|^{\beta _2}} \, ds \, d\kappa \, d{\tilde{\kappa }}. \end{aligned}$$

Suppose that we can show that

$$\begin{aligned} {\mathbb {E}}[M_{1*}] \lesssim y^r, \quad {\mathbb {E}}[M_{2j}] \lesssim y^r \end{aligned}$$

for some $r>0$. This would imply that they are almost surely finite, and that *G* and ${\hat{G}}$ are Hölder continuous with $\Vert G\Vert _{C^{\alpha ,\eta }} \lesssim M_{A*}^{1/\lambda }+M_{21}^{1/\lambda }+M_{22}^{1/p}$ (same for ${\hat{G}}$).

Notice that now $A_{1*}, A_{21}, A_{22}$, hence also $M_{A*}, M_{21}, M_{22}$ are decreasing in *y*. So as we let $y,y_1 \searrow 0$, it would follow that

- ${\mathbb {E}}[\Vert G\Vert _{C^{\alpha ,\eta }}^\lambda ] \lesssim y^{r'} \rightarrow 0$ (same for ${\hat{G}}$) with a (possibly) different $r'>0$. In particular, as $y \searrow 0$, the random functions $v(\cdot ,\cdot ,y)$ and $(t,\kappa ) \mapsto {\hat{f}}^\kappa _t(iy)$ form Cauchy sequences in $L^\lambda ({\mathbb {P}};C^{\alpha ,\eta })$, and it follows that also ${\mathbb {E}}[\Vert v(\cdot ,\cdot ,y)\Vert _{C^{\alpha ,\eta }}^\lambda ] \lesssim y^{r'} \rightarrow 0$ and ${\mathbb {E}}[\Vert \gamma (\cdot ,\cdot ) - {\hat{f}}^\cdot _\cdot (iy)\Vert _{C^{\alpha ,\eta }}^\lambda ] \lesssim y^{r'} \rightarrow 0$ as $y \searrow 0$.
- By the monotonicity of $M_{A*}, M_{21}, M_{22}$ in *y* we have that almost surely the functions $v(\cdot ,\cdot ,y)$ and $(t,\kappa ) \mapsto {\hat{f}}^\kappa _t(iy)$ are Cauchy sequences in the Hölder space $C^{\alpha ,\eta }$.

This will show Theorem 4.1.

**Step 2** We now explain that in fact, our definition of $A_{1*}$ does not always suffice, and we need to define $A_{1j}$ a bit differently in order to get the best estimates. The new definition of $A_{1j}$ will satisfy only the relaxed condition (14) [instead of (5)].

The reason is that, when $|t-s| \le u^2$, $|{\hat{f}}_t(iu)-{\hat{f}}_s(iu)|$ is estimated by an expression like $|{\hat{f}}_s'(iu)| |B_t-B_s|$ which is of the order $O(|t-s|^{1/2})$. The same is true for the difference $|{\hat{f}}_t'(iu)-{\hat{f}}_s'(iu)|$ [see (20) below]. When we carry out the moment estimate for our choice of $A_{1*}$, then we will get

$$\begin{aligned} {\mathbb {E}}|A_{1*}(t,s;\kappa )|^\lambda = O(|t-s|^{\lambda /2}). \end{aligned}$$

But recall from Proposition 3.3 that

$$\begin{aligned} {\mathbb {E}}|\gamma (t)-\gamma (s)|^\lambda \le C|t-s|^{(\zeta +\lambda )/2}, \end{aligned}$$

which has allowed us to apply Lemma 2.1 with $\beta _1 \approx \frac{\zeta +\lambda }{2}+1$ in the proof of Theorem 3.2. When $\zeta >0$, this was better than just $\lambda /2$.

To fix this, we need to adjust our choice of $A_{1j}$. In particular, we should not evaluate ${\mathbb {E}}|{\hat{f}}_t'(iu)-{\hat{f}}_s'(iu)|^\lambda$ when $u \gg |t-s|^{1/2}$ (here “$\gg$” means “much larger”). As observed in [9], $|{\hat{f}}_s'(iu)|$ does not change much in time when $u \gg |t-s|^{1/2}$. More precisely, we have the following results.

### Lemma 4.5

Let $(g_t)$ be a chordal Loewner chain driven by *U*, and ${\hat{f}}_t(z) = g_t^{-1}(z+U(t))$. Then, if $t,s \ge 0$ and $z=x+iy \in {\mathbb {H}}$ such that $|t-s| \le C' y^2$, we have

18 $$\begin{aligned} |{\hat{f}}_t'(z)|&\le C|{\hat{f}}_s'(z)| \left( 1+\frac{|U(t)-U(s)|^2}{y^2} \right) ^l, \end{aligned}$$

19 $$\begin{aligned} |{\hat{f}}_t(z)-{\hat{f}}_s(z)|&\le C |{\hat{f}}_s'(z)| \left( \frac{|t-s|}{y} + |U(t)-U(s)| \left( 1+\frac{|U(t)-U(s)|^2}{y^2} \right) ^l \right) , \end{aligned}$$

20 $$\begin{aligned} |{\hat{f}}_t'(z)-{\hat{f}}_s'(z)|&\le C |{\hat{f}}_s'(z)| \left( \frac{|t-s|}{y^2} + \frac{|U(t)-U(s)|}{y} \left( 1+\frac{|U(t)-U(s)|^2}{y^2} \right) ^l \right) , \end{aligned}$$

where $C < \infty$ depends on $C' < \infty$, and $l < \infty$ is a universal constant.

### Proof

The first two inequalities (18) and (19) follow from [9, Lemma 3.5 and 3.2]. The third inequality (20) follows from (19) by the Cauchy integral formula in the same way as in Corollary 3.7. Note that for $z \in {\mathbb {H}}$ and *w* on a circle of radius *y*/2 around *z*, we have $|{\hat{f}}_s'(w)| \le 12 |{\hat{f}}_s'(z)|$ by the Koebe distortion theorem. $\square$

We now redefine $A_{1j}$. Let

$$\begin{aligned} A_{11}(t,s;\kappa )&= \int _0^{y \wedge |t-s|^{1/2}} |{\hat{f}}_t'(iu)-{\hat{f}}_s'(iu)| \, du,\\ A_{12}(t,s;\kappa )&= \int _{y \wedge |t-s|^{1/2}}^y \frac{|t-s|}{u^2} |{\hat{f}}_s'(iu)| \, du,\\ A_{13}(t,s;\kappa )&= \int _{y \wedge |t-s|^{1/2}}^{y \wedge 2|t-s|^{1/2}} u^{-1} |{\hat{f}}_s'(iu)| \left( 1+\Vert B\Vert _{C^{1/2^{(-)}}}\right) ^{2l+1} |t-s|^{1/2^{(-)}} \, du, \end{aligned}$$

for $s \le t$, where the exponents $1/2^{(-)} < 1/2$ denote some numbers that we can pick arbitrarily close to 1/2. (Of course, ${\hat{f}}_t$ still depends on $\kappa$, but for convenience we do not write it for now.)

Note that the integrands in $A_{12}$ and $A_{13}$ just make fancy bounds of

$$\begin{aligned} |{\hat{f}}_t'(iu)-{\hat{f}}_s'(iu)|, \end{aligned}$$

according to (20). But now, in $A_{13}$ we are not integrating up to *y* any more. Thus, the condition (5) is not satisfied any more. But the relaxed condition (14) of Lemma 2.13 is still satisfied. Indeed, by (20),

$$\begin{aligned} \begin{aligned} A_{1*}(t,s;\kappa )&\le A_{11}(t,s;\kappa ) + \int _{y \wedge |t-s|^{1/2}}^y |{\hat{f}}_t'(iu)-{\hat{f}}_s'(iu)| \, du \\&\le A_{11}(t,s;\kappa ) + A_{12}(t,s;\kappa ) \\&\quad +\, \int _{y \wedge |t-s|^{1/2}}^y u^{-1} |{\hat{f}}_s'(iu)| \left( 1+\Vert B\Vert _{C^{1/2^{(-)}}}\right) ^{l+1} |t-s|^{1/2^{(-)}} \, du \end{aligned} \end{aligned}$$

where by (18)

$$\begin{aligned} \begin{aligned}&\int _{y \wedge |t-s|^{1/2}}^y u^{-1} |{\hat{f}}_s'(iu)| \left( 1+\Vert B\Vert _{C^{1/2^{(-)}}}\right) ^{l+1} |t-s|^{1/2^{(-)}} \, du \\&\quad = \sum _{k=0}^{\lfloor \log _4(y^2/|t-s|) \rfloor } \int _{y \wedge (4^k|t-s|)^{1/2}}^{y \wedge 2(4^k|t-s|)^{1/2}} \ldots \\&\quad = \sum _{k=0}^{\lfloor \log _4(y^2/|t-s|) \rfloor } 4^{-k(1/2^{(-)})} |A_{13}(t_1+4^k(t-t_1),t_1+4^k(s-t_1);\kappa )| \end{aligned} \end{aligned}$$

whenever $$|s-t_1| \le 2|t-s|$$ (implying $$|s-(t_1+4^k(s-t_1))| \le (4^k-1)2|t-s| \le 2u^2$$).

Finally, with this definition of $A_{13}$, we truly have ${\mathbb {E}}|A_{13}(t,s;\kappa )|^{\lambda ^{(-)}} = O(|t-s|^{(\zeta +\lambda )^{(-)}/2})$ and not just $O(|t-s|^{\lambda /2})$; here $\lambda ^{(-)} < \lambda$ is an exponent that can be chosen arbitrarily close to $\lambda$.

### Proposition 4.6

With the above notation and assumptions, if $1< \beta _1 < \frac{\zeta +\lambda }{2}+1$, $1< \beta _2 < p+1$, we have

$$\begin{aligned} {\mathbb {E}}\iiint \frac{|A_{1j}(t,s;\kappa )|^\lambda }{|t-s|^{\beta _1}} \, ds \, dt \, d\kappa&\le C y^{\zeta +\lambda -2\beta _1+2} \iint a(s,\zeta _\kappa ) \, ds \, d\kappa , \quad j=1,2,\\ {\mathbb {E}}\iiint \frac{|A_{13}(t,s;\kappa )|^{\lambda ^{(-)}}}{|t-s|^{\beta _1}} \, ds \, dt \, d\kappa&\le C y^{(\zeta +\lambda )^{(-)} -2\beta _1+2} \iint a(s,\zeta _\kappa )^{1^{(-)}} \, ds \, d\kappa ,\\ {\mathbb {E}}\iiint \frac{|A_{21}(s;\kappa ,{\tilde{\kappa }})|^\lambda }{|\kappa -{\tilde{\kappa }}|^{\beta _2}} \, ds \, d\kappa \, d{\tilde{\kappa }}&\le C y^{(\zeta +\lambda )(p-\beta _2+1)/p} \iint a(s,\zeta _\kappa ) \, ds \, d\kappa ,\\ {\mathbb {E}}\iiint \frac{|A_{22}(s;\kappa ,{\tilde{\kappa }})|^p}{|\kappa -{\tilde{\kappa }} |^{\beta _2}} \, ds \, d\kappa \, d{\tilde{\kappa }}&\le C y^{(\zeta +\lambda )(p-\beta _2+1)/p}, \end{aligned}$$

where *C* depends on $\kappa _-$, $\kappa _+$, $\lambda$, *p*, $\beta _1$, $\beta _2$.

### Proof

These follow from direct computations making use of Lemma 3.1 and Corollary 3.7. They can be found in the appendix of the arXiv version of this paper. $\square$

Recall that the condition for Lemma 2.1 is $(\beta _1-2)(\beta _2-2)-1 > 0$. With $\beta _1 < \frac{\lambda +\zeta }{2}+1$, $\beta _2 < p+1$ this is again the condition $(\frac{\zeta +\lambda }{2})^{-1}+p^{-1} < 1$, which leads to $\kappa < \frac{8}{3}$. Moreover, we need the additional condition $\frac{\beta _1-2}{\lambda } < 1/2^{(-)}$ for Lemma 2.13, which is implied by $\zeta < 2$.

The same analysis of $\lambda$ and $\zeta$ as in the proof of Theorem 3.2 applies here. This finishes the proof of Theorem 4.1. $\square$

## Proof of Proposition 3.5

The proof is based on the methods of [10, 15].

Let $t \ge 0$ and $U \in C([0,t];{\mathbb {R}})$. We study the chordal Loewner chain $(g_s)_{s \in [0,t]}$ in ${\mathbb {H}}$ driven by *U*, i.e. the solution of (15). Let $V(s) = U(t-s)-U(t)$, $s \in [0,t]$, and consider the solution of the reverse flow

21 $$\begin{aligned} \partial _s h_s(z) = \frac{-2}{h_s(z)-V(s)}, \quad h_0(z) = z. \end{aligned}$$

The Loewner equation implies $h_t(z) = g_t^{-1}(z+U(t))-U(t) = {\hat{f}}_t(z)-U(t)$.

Let $x_s + iy_s = z_s = z_s(z) = h_s(z)-V(s)$. Recall that

$$\begin{aligned} \partial _s \log |h_s'(z)| = 2 \frac{x_s^2-y_s^2}{(x_s^2+y_s^2)^2} \end{aligned}$$

and therefore

$$\begin{aligned} |h_s'(z)| = \exp \left( 2 \int _0^s \frac{x_\vartheta ^2-y_\vartheta ^2}{(x_\vartheta ^2+y_\vartheta ^2)^2} \, d\vartheta \right) . \end{aligned}$$

For $r \in [0,t]$, denote by $h_{r,s}$ the reverse Loewner flow driven by $V(s)-V(r)$, $s \in [r,t]$. More specifically,

$$\begin{aligned} \partial _s (h_{r,s}(z_r(z))+V(r))&= \frac{-2}{(h_{r,s}(z_r(z))+V(r))-V(s)},\\ h_{r,r}(z_r(z))+V(r)&= z_r(z)+V(r) = h_r(z), \end{aligned}$$

which implies from (21) that

$$\begin{aligned}&h_{r,s}(z_r(z))+V(r) = h_s(z)\\&\quad \text {and}\, z_{r,s}(z_r(z)) = z_s(z) \quad \text {for all } s \in [r,t]. \end{aligned}$$

This implies also

$$\begin{aligned} |h_{r,s}'(z_r(z))| = \exp \left( 2 \int _r^s \frac{x_\vartheta ^2-y_\vartheta ^2}{(x_\vartheta ^2+y_\vartheta ^2)^2} \, d\vartheta \right) . \end{aligned}$$

The following result is essentially [10, Lemma 2.3], stated in a more refined way.

### Lemma 5.1

Let $V^1, V^2 \in C([0,t];{\mathbb {R}})$, and denote by $(h^j_s)$ the reverse Loewner flow driven by $V^j$, $j=1,2$, respectively. For $z=x+iy$, denoting $x^j_s + iy^j_s = z^j_s = h^j_s(z)-V^j(s)$, we have

$$\begin{aligned}&|h^1_t(z)-h^2_t(z)| \\&\quad \le 2(y^2+4t)^{1/4} \int _0^t |V^1(s)-V^2(s)| \frac{1}{|z^1_s z^2_s|} \frac{1}{(y^1_s y^2_s)^{1/4}} |(h^1_{s,t})'(z^1_s) (h^2_{s,t})'(z^2_s)|^{1/4} \, ds. \end{aligned}$$

### Proof

The proof of [10, Lemma 2.3] shows that

$$\begin{aligned}&|h^1_t(z)-h^2_t(z)| \\&\quad \le \int _0^t |V^1(s)-V^2(s)| \frac{2}{|z^1_s z^2_s|} \exp \left( 2 \int _s^t \frac{x^1_\vartheta x^2_\vartheta - y^1_\vartheta y^2_\vartheta }{((x^1_\vartheta )^2+(y^1_\vartheta )^2) ((x^2_\vartheta )^2+(y^2_\vartheta )^2)} \, d\vartheta \right) \, ds. \end{aligned}$$

The claim follows by estimating

$$\begin{aligned}&2 \int _s^t \frac{x^1_\vartheta x^2_\vartheta - y^1_\vartheta y^2_\vartheta }{((x^1_\vartheta )^2+(y^1_\vartheta )^2)((x^2_\vartheta )^2+(y^2_\vartheta )^2)} \, d\vartheta \\&\quad \le 2 \int _s^t \frac{x^1_\vartheta x^2_\vartheta }{((x^1_\vartheta )^2+(y^1_\vartheta )^2)((x^2_\vartheta )^2+(y^2_\vartheta )^2)} \, d\vartheta \\&\quad \le \prod _{j=1,2} \left( 2 \int _s^t \frac{(x^j_\vartheta )^2}{((x^j_\vartheta )^2+(y^j_\vartheta )^2)^2} \, d\vartheta \right) ^{1/2}\\&\quad = \prod _{j=1,2} \left( \frac{1}{2} \int _s^t \frac{2((x^j_\vartheta )^2-(y^j_\vartheta )^2)}{((x^j_\vartheta )^2+(y^j_\vartheta )^2)^2} \, d\vartheta + \frac{1}{2} \int _s^t \frac{2}{(x^j_\vartheta )^2+(y^j_\vartheta )^2} \, d\vartheta \right) ^{1/2}\\&\quad = \prod _{j=1,2} \left( \frac{1}{2} \log |(h^j_{s,t})'(z^j_s)| + \frac{1}{2} \log \frac{y^j_t}{y^j_s} \right) ^{1/2}\\&\quad \le \sum _{j=1,2} \left( \frac{1}{4} \log |(h^j_{s,t})'(z^j_s)| + \frac{1}{4} \log \frac{y^j_t}{y^j_s} \right) \end{aligned}$$

and $y^j_t \le \sqrt{y^2+4t}$. (In the last line we used $\sqrt{ab}\le \frac{a+b}{2}$ for $a,b\ge 0$.) $\square$

### Taking moments

Let $\kappa ,{\tilde{\kappa }} > 0$, and let $V^1 = \sqrt{\kappa }B$, $V^2 = \sqrt{{\tilde{\kappa }}}B$, where *B* is a standard Brownian motion. In the following, *C* will always denote a finite deterministic constant that might change from line to line.

Lemma 5.1 and the Cauchy–Schwarz inequality imply

22 $$\begin{aligned}&{\mathbb {E}}|h^1_t(z)-h^2_t(z)|^p \nonumber \\&\quad \le C |\Delta \sqrt{\kappa }|^p \, {\mathbb {E}}\left| \int _0^t |B_s| \frac{1}{|z^1_s z^2_s|} \frac{1}{(y^1_s y^2_s)^{1/4}} |(h^1_{s,t})'(z^1_s) (h^2_{s,t})'(z^2_s)|^{1/4} \, ds \right| ^p \nonumber \\&\quad \le C |\Delta \sqrt{\kappa }|^p \, {\mathbb {E}}\prod _{j=1,2} \left| \int _0^t |B_s| \frac{1}{|z^j_s|^2} \frac{1}{(y^j_s)^{1/2}} |(h^j_{s,t})'(z^j_s)|^{1/2} \, ds \right| ^{p/2} \nonumber \\&\quad \le C |\Delta \sqrt{\kappa }|^p \prod _{j=1,2} \left( {\mathbb {E}}\left| \int _0^t |B_s| \frac{1}{|z^j_s|^2} \frac{1}{(y^j_s)^{1/2}} |(h^j_{s,t})'(z^j_s)|^{1/2} \, ds \right| ^p \right) ^{1/2}. \end{aligned}$$

Now the flows for $\kappa$ and ${\tilde{\kappa }}$ can be studied separately. We see that as long as the above integral is bounded, then ${\mathbb {E}}|\Delta _{\sqrt{\kappa }} h^\kappa _t(z)|^p \lesssim |\Delta \sqrt{\kappa }|^p$. Heuristically, the typical growth of $y_s$ is like $\sqrt{s}$, as was shown in [15]. Therefore, we expect the integrand to be bounded by $s^{1/2-1-1/4-\beta /4} = s^{-(3+\beta )/4}$ which is integrable since $\beta = \beta (\kappa ) < 1$ for $\kappa \ne 8$.

In order to make the idea precise, we will reparametrise the integral in order to match the setting in [15] and apply their results.

### Reparametrisation

Let $\kappa > 0$. In [15], the flow

23 $$\begin{aligned} \partial _s {\tilde{h}}_s(z) = \frac{-a}{{\tilde{h}}_s (z)-{\tilde{B}}_s}, \quad {\tilde{h}}_0(z) = z, \end{aligned}$$

with $a = \dfrac{2}{\kappa }$ is considered. To translate our notation, observe that

$$\begin{aligned} \partial _s h_{s/\kappa }(z) = \frac{-2/\kappa }{h_{s/\kappa } (z)-\sqrt{\kappa }B_{s/\kappa }}. \end{aligned}$$

If we let ${\tilde{B}}_s = \sqrt{\kappa }B_{s/\kappa }$, then

$$\begin{aligned} h_{s/\kappa }(z) = {\tilde{h}}_s(z) \implies h_s(z) = {\tilde{h}}_{\kappa s}(z). \end{aligned}$$

Moreover, if we let ${\tilde{z}}_s = {\tilde{h}}_s(z) - {\tilde{B}}_s$, then $z_s = h_s(z) - \sqrt{\kappa }B_s = {\tilde{z}}_{\kappa s}$.

Therefore,

$$\begin{aligned} \int _0^t |B_s| \frac{1}{|z_s|^2} \frac{1}{y_s^{1/2}} |h_{s,t}'(z_s)|^{1/2} \, ds&= \int _0^t \left| \frac{1}{\sqrt{\kappa }}{\tilde{B}}_{\kappa s} \right| \frac{1}{|{\tilde{z}}_{\kappa s}|^2} \frac{1}{{\tilde{y}}_{\kappa s}^{1/2}} |{\tilde{h}}_{\kappa s,\kappa t}'({\tilde{z}}_{\kappa s})|^{1/2} \, ds\\&= \int _0^{\kappa t} \kappa ^{-3/2} |{\tilde{B}}_s| \frac{1}{|{\tilde{z}}_s|^2} \frac{1}{{\tilde{y}}_s^{1/2}} |{\tilde{h}}_{s,\kappa t}'({\tilde{z}}_s)|^{1/2} \, ds. \end{aligned}$$

For notational simplicity, we will write just *t* instead of $\kappa t$ and $B, h_s, z_s$ instead of ${\tilde{B}}, {\tilde{h}}_s, {\tilde{z}}_s$.

In the next step, we will let the flow start at $z_0 = i$ instead of $i\delta$. Observe that

$$\begin{aligned} \partial _s (\delta ^{-1} h_{\delta ^2 s}(\delta z)) = \frac{-a}{\delta ^{-1} h_{\delta ^2 s}(\delta z) - \delta ^{-1} B_{\delta ^2 s}}, \end{aligned}$$

so we can write $h_s(\delta z) = \delta {\tilde{h}}_{s/\delta ^2}(z)$ where $({\tilde{h}}_s)$ is driven by $\delta ^{-1} B_{\delta ^2 s} =: {\tilde{B}}_s$. Note that ${\tilde{h}}_{s/\delta ^2}'(z) = h_s'(\delta z)$. As before, we denote $z_s = h_s(\delta z)-B_s$ and ${\tilde{z}}_s = {\tilde{h}}_s(z)-{\tilde{B}}_s$, where $z_s = \delta {\tilde{z}}_{s/\delta ^2}$. Consequently,

$$\begin{aligned}&\int _0^t |B_s| \frac{1}{|z_s|^2} \frac{1}{y_s^{1/2}} |h_{s,t}'(z_s)|^{1/2} \, ds \\&\quad = \int _0^t |\delta {\tilde{B}}_{s/\delta ^2}| \frac{1}{\delta ^2 |{\tilde{z}}_{s/\delta ^2}|^2} \frac{1}{\delta ^{1/2} {\tilde{y}}_{s/\delta ^2}^{1/2}} |{\tilde{h}}_{s/\delta ^2,t/\delta ^2}'({\tilde{z}}_{s/\delta ^2})|^{1/2} \, ds\\&\quad = \delta ^{-3/2} \int _0^t |{\tilde{B}}_{s/\delta ^2}| \frac{1}{|{\tilde{z}}_{s/\delta ^2}|^2} \frac{1}{{\tilde{y}}_{s/\delta ^2}^{1/2}} |{\tilde{h}}_{s/\delta ^2,t/\delta ^2}'({\tilde{z}}_{s/\delta ^2})|^{1/2} \, ds\\&\quad = \delta ^{1/2} \int _0^{t/\delta ^2} |{\tilde{B}}_s| \frac{1}{|{\tilde{z}}_s|^2} \frac{1}{{\tilde{y}}_s^{1/2}} |{\tilde{h}}_{s,t/\delta ^2}'({\tilde{z}}_s)|^{1/2} \, ds. \end{aligned}$$

Again, for notational simplicity we will stop writing the $\tilde{\ }$ from now on.

Now, let $z_0 = i$, and (cf. [15])

$$\begin{aligned} \sigma (s) = \inf \{ r \mid y_r = e^{ar}\} = \int _0^s |z_{\sigma (r)}|^2 \, dr \end{aligned}$$

which is random and strictly increasing in *s*.

Then

$$\begin{aligned}&\delta ^{1/2} \int _0^{t/\delta ^2} |B_s| \frac{1}{|z_s|^2} \frac{1}{y_s^{1/2}} |h_{s,t/\delta ^2}'(z_s)|^{1/2} \, ds \\&\quad = \delta ^{1/2} \int _0^{\sigma ^{-1}(t/\delta ^2)} |B_{\sigma (s)}| \frac{1}{y_{\sigma (s)}^{1/2}} |h_{{\sigma (s)},t/\delta ^2}'(z_{\sigma (s)})|^{1/2} \, ds. \end{aligned}$$

This is the integral we will work with.

To sum it up, we have the following.

#### Proposition 5.2

Let $z \in {\mathbb {H}}$, and $(h_s(\delta z))_{s \ge 0}$ satisfy (21) with $V(s) = \sqrt{\kappa } B_s$ and a standard Brownian motion *B*, and $({\tilde{h}}_s(z))_{s \ge 0}$ satisfy (23) with a standard Brownian motion ${\tilde{B}}$. Let $x_s + iy_s = z_s = h_s(\delta z)-V(s)$, and ${\tilde{x}}_s + i{\tilde{y}}_s = {\tilde{z}}_s = {\tilde{h}}_s(z) - {\tilde{B}}_s$. Then, with the notations above,

$$\begin{aligned} \int _0^t |B_s| \frac{1}{|z_s|^2} \frac{1}{y_s^{1/2}} |h_{s,t}'(z_s)|^{1/2} \, ds \end{aligned}$$

has the same law as

$$\begin{aligned} \kappa ^{-3/2} \delta ^{1/2} \int _0^{\sigma ^{-1}(\kappa t/\delta ^2)} |{\tilde{B}}_{\sigma (s)}| \frac{1}{{\tilde{y}}_{\sigma (s)}^{1/2}} |{\tilde{h}}_{{\sigma (s)},\kappa t/\delta ^2}'({\tilde{z}}_{\sigma (s)})|^{1/2} \, ds. \end{aligned}$$

(Recall that $${\tilde{y}}_{\sigma (s)}= e^{as}.)$$

### Main proof

In the following, we fix $\kappa \in [\kappa _-,\kappa _+]$, $a = \dfrac{2}{\kappa }$, and let $(h_s(x+i))_{s \ge 0}$ satisfy (23) with initial point $z_0 = x+i$, $|x| \le 1$.

Our goal is to estimate

$$\begin{aligned}&{\mathbb {E}}\left| \delta ^{1/2} \int _0^{\sigma ^{-1} (t/\delta ^2)} |B_{\sigma (s)}| \frac{1}{y_{\sigma (s)}^{1/2}} |h_{{\sigma (s)},t/\delta ^2}'(z_{\sigma (s)})|^{1/2} \, ds \right| ^p\\&\quad = {\mathbb {E}}\left| \delta ^{1/2} \int _0^\infty 1_{\sigma (s) \le t/\delta ^2} |B_{\sigma (s)}| \frac{1}{y_{\sigma (s)}^{1/2}} |h_{{\sigma (s)},t/\delta ^2}'(z_{\sigma (s)})|^{1/2} \, ds \right| ^p. \end{aligned}$$

With (22) and Proposition 5.2 this will complete the proof of Proposition 3.5.

From the definition of $\sigma$ it follows that $\sigma (s) \ge \int _0^s e^{2ar} \, dr = \frac{1}{2a}(e^{2as}-1)$, or equivalently, $\sigma ^{-1}(t) \le \frac{1}{2a}\log (1+2at)$. Therefore, $\sigma ^{-1}(t/\delta ^2) \le \frac{1}{a}\log \frac{C}{\delta }$ and

24 $$\begin{aligned}&{\mathbb {E}}\left| \delta ^{1/2} \int _0^{\sigma ^{-1}(t/\delta ^2)} |B_{\sigma (s)}| \frac{1}{y_{\sigma (s)}^{1/2}} |h_{{\sigma (s)},t/\delta ^2}'(z_{\sigma (s)})|^{1/2} \, ds \right| ^p\nonumber \\&\quad \le \delta ^{p/2} \left( \int _0^{\frac{1}{a}\log \frac{C}{\delta }} \left( {\mathbb {E}}\left[ 1_{\sigma (s) \le t/\delta ^2} |B_{\sigma (s)}|^p \frac{1}{y_{\sigma (s)}^{p/2}} |h_{{\sigma (s)},t/\delta ^2}'(z_{\sigma (s)})|^{p/2} \right] \right) ^{1/p} \, ds \right) ^p\nonumber \\ \end{aligned}$$

where we have applied Minkowski’s inequality to pull the moment inside the integral.

To proceed, we need to know more about the behaviour of the reverse SLE flow, which also incorporates the behaviour of $\sigma$. This has been studied in [15]. Their tool was to study the process $J_s$ defined by $\sinh J_s = \frac{x_{\sigma (s)}}{y_{\sigma (s)}} = e^{-as}x_{\sigma (s)}$. By [15, Lemma 6.1], this process satisfies

$$\begin{aligned} dJ_s = -r_c \tanh J_s \, ds + dW_s \end{aligned}$$

where $W_s = \int _0^{\sigma (s)} \frac{1}{|z_r|} \, dB_r$ is a standard Brownian motion and $r_c$ is defined in (17).

The following results have been originally stated for an equivalent probability measure ${\mathbb {P}}_*$, depending on a parameter *r*, such that

$$\begin{aligned} dJ_s = -q \tanh J_s \, ds + dW^*_s \end{aligned}$$

with $q>0$ and a process $W^*$ that is a Brownian motion under ${\mathbb {P}}_*$. But setting the parameter $r=0$, we have ${\mathbb {P}}_* = {\mathbb {P}}$, $q=r_c$, and $W^* = W$. Therefore, under the measure ${\mathbb {P}}$, the results apply with $q=r_c$.

Note also that although the results were originally stated for a reverse SLE flow starting at $z_0 = i$, they can be written for flows starting at $z_0 = x+i$ without change of the proof. One just uses [15, Lemma 7.1 (28)] with $\cosh J_0 = \sqrt{1+x^2}$.

Recall that [9, 15] use the notation $\sinh J_s = \frac{x_{\sigma (s)}}{y_{\sigma (s)}}$ and hence $\cosh ^2 J_s = 1+\frac{x_{\sigma (s)}^2}{y_{\sigma (s)}^2}$.

#### Lemma 5.3

[9, Lemma 5.6] Suppose $z_0 = x+i$. There exists a constant $C < \infty$, depending on $\kappa _-$, $\kappa _+$, such that for each $s \ge 0$, $u > 0$ there exists an event $E_{u,s}$ with

$$\begin{aligned} {\mathbb {P}}(E_{s,u}^c) \le C (1+x^2)^{r_c} u^{-2r_c} \end{aligned}$$

on which

$$\begin{aligned} \sigma (s) \le u^2 e^{2as} \quad \text {and} \quad 1+\frac{x_{\sigma (s)}^2}{y_{\sigma (s)}^2} \le u^2/4. \end{aligned}$$

Fix $$s \in [0,t]$$. Let

$$\begin{aligned} E_u = \left\{ \sigma (s) \le u^2 e^{2as} \text { and } 1+\frac{x_{\sigma (s)}^2}{y_{\sigma (s)}^2} \le u^2 \right\} \end{aligned}$$

and $A_n$ = $E_{\exp (n)} {\setminus } E_{\exp (n-1)}$ for $n \ge 1$, and $A_0 = E_1$. Then

25 $$\begin{aligned} {\mathbb {P}}(A_n) \le {\mathbb {P}}(E_{\exp (n-1)}^c) \le C (1+x^2)^{r_c} e^{-2r_c n}. \end{aligned}$$

(The constant *C* may change from line to line.)

#### Lemma 5.4

(see proof of [9, Lemma 5.7]) Suppose $z_0 = x+i$. There exists $C < \infty$, depending on $\kappa _-$, and a global constant $\alpha > 0$, such that for all $s \ge 0$, $u > \sqrt{1+x^2}$, and $k > 2a$ we have

$$\begin{aligned} {\mathbb {P}}\left( \sigma (s) \le u^2 e^{2as} \text { and } 1+\frac{x_{\sigma (s)}^2}{y_{\sigma (s)}^2} \ge u^2 e^k \right) \le C (1+x^2)^{r_c} u^{-2r_c} e^{-\alpha (k-2a)^2}. \end{aligned}$$

We proceed to estimating

26 $$\begin{aligned}&{\mathbb {E}}\left[ 1_{A_n} 1_{\sigma (s) \le t/\delta ^2} |B_{\sigma (s)}|^p \frac{1}{y_{\sigma (s)}^{p/2}} |h_{{\sigma (s)},t/\delta ^2}'(z_{\sigma (s)})|^{p/2} \right] \nonumber \\&\quad = {\mathbb {E}}\left[ 1_{A_n} 1_{\sigma (s) \le t/\delta ^2} |B_{\sigma (s)}|^p \frac{1}{y_{\sigma (s)}^{p/2}} {\mathbb {E}}\left[ |h_{{\sigma (s)},t/\delta ^2}'(z_{\sigma (s)})|^{p/2} \mid {\mathcal {F}}_{\sigma (s)} \right] \right] \end{aligned}$$

where ${\mathcal {F}}$ is the filtration generated by *B*.

Note that $y_{\sigma (s)} = e^{as}$ by the definition of $\sigma$. Moreover, on the set $A_n$, the Brownian motion is easy to handle since by Hölder’s inequality

27 $$\begin{aligned} {\mathbb {E}}[ 1_{A_n} 1_{\sigma (s) \le t/\delta ^2} |B_{\sigma (s)}|^p ]&\le {\mathbb {E}}\left[ 1_{A_n} 1_{\sigma (s) \le t/\delta ^2} \sup _{r \in [0, e^{2n} e^{2as}]} |B_r|^p \right] \nonumber \\&\le {\mathbb {P}}(A_n \cap \{\sigma (s) \le t/\delta ^2\})^{1-\varepsilon }\, {\mathbb {E}}\left[ \sup _{r \in [0, e^{2n} e^{2as}]} |B_r|^{p/\varepsilon } \right] ^{\varepsilon } \nonumber \\&\le C\, {\mathbb {P}}(A_n \cap \{\sigma (s) \le t/\delta ^2\})^{1-\varepsilon }\, e^{np} e^{pas} \end{aligned}$$

for any $$\varepsilon > 0$$.

It remains to handle ${\mathbb {E}}\left[ |h_{{\sigma (s)},t/\delta ^2}'(z_{\sigma (s)})|^{p/2} \mid {\mathcal {F}}_{\sigma (s)} \right]$.

The following result is well-known and follows from the Schwarz lemma and mapping the unit disc to the half-plane.

#### Lemma 5.5

Let $f{:}\,{\mathbb {H}}\rightarrow {\mathbb {H}}$ be a holomorphic function. Then $|f'(z)| \le \frac{\mathfrak {I}(f(z))}{\mathfrak {I}(z)}$ for all $z \in {\mathbb {H}}$.

Recall that the Loewner equation implies

$$\begin{aligned} \mathfrak {I}(h_{{\sigma (s)},t/\delta ^2}(z_{\sigma (s)})) = y_{t/\delta ^2} \le \sqrt{1+2at/\delta ^2} \le C\delta ^{-1}. \end{aligned}$$

Let $\varepsilon > 0$. By the lemma above, we can estimate

28 $$\begin{aligned}&{\mathbb {E}}\left[ |h_{{\sigma (s)},t/\delta ^2}'(z_{\sigma (s)})|^{p/2} \mid {\mathcal {F}}_{\sigma (s)} \right] \nonumber \\&\quad \le (\delta y_{\sigma (s)})^{-(1-\varepsilon )p/2} {\mathbb {E}}\left[ |h_{{\sigma (s)},t/\delta ^2}'(z_{\sigma (s)})|^{\varepsilon p/2} \mid {\mathcal {F}}_{\sigma (s)} \right] . \end{aligned}$$

From [9, Lemma 3.2] it follows that there exists some $l>0$ such that

29 $$\begin{aligned} |h_{{\sigma (s)},t/\delta ^2}'(z_{\sigma (s)})| \le C\left( 1+\frac{x_{\sigma (s)}^2}{y_{\sigma (s)}^2}\right) ^l |h_{{\sigma (s)},t/\delta ^2}'(iy_{\sigma (s)})|. \end{aligned}$$

We claim that

30 $$\begin{aligned} {\mathbb {E}}\left[ |h_{{\sigma (s)},t/\delta ^2}'(iy_{\sigma (s)})|^{\varepsilon p/2} \mid {\mathcal {F}}_{\sigma (s)} \right] \le C \end{aligned}$$

if $\varepsilon >0$ is sufficiently small.

To see this, first recall that for small $\varepsilon > 0$ we have

31 $$\begin{aligned} {\mathbb {E}}\left[ |h_t'(i)|^\varepsilon \right] \le C \end{aligned}$$

uniformly in $t \ge 1$. This follows from [9, Theorem 5.4] or, even more elementary, from the proof of [18, Theorem 3.2].

Now approximate $\sigma (s)$ by simple stopping times ${\tilde{\sigma }} \ge \sigma (s)$. A possible choice is ${\tilde{\sigma }} = \lceil \sigma (s) 2^n \rceil 2^{-n} \wedge t/\delta ^2$. It suffices to show

$$\begin{aligned} {\mathbb {E}}\left[ |h_{{{\tilde{\sigma }}},t/\delta ^2}'(iy_{\sigma (s)})|^{\varepsilon p/2} \mid {\mathcal {F}}_{\sigma (s)} \right] \le C \end{aligned}$$

and then apply Fatou’s lemma to pass to the limit.

Now that ${\tilde{\sigma }}$ is simple, we can apply (31) on each set $F_r = \{ {\tilde{\sigma }} = r \}$. Using the strong Markov property of Brownian motion and the scaling invariance of SLE, we get

$$\begin{aligned} {\mathbb {E}}\left[ 1_{F_r} |h_{{{\tilde{\sigma }}},t/\delta ^2}'(ie^{as})|^{\varepsilon p/2} \mid {\mathcal {F}}_{\sigma (s)} \right]&= 1_{F_r} {\mathbb {E}}\left[ |h_{r,t/\delta ^2}'(ie^{as})|^{\varepsilon p/2} \right] \\&= 1_{F_r} {\mathbb {E}}\left[ |h_{e^{-2as}(t/\delta ^2-r)}'(i)|^{\varepsilon p/2} \right] \\&\le 1_{F_r} C \end{aligned}$$

and the claim follows.

Combining (28)–(30), we have

32 $$\begin{aligned} {\mathbb {E}}\left[ |h_{{\sigma (s)},t/\delta ^2}'(z_{\sigma (s)})|^{p/2} \mid {\mathcal {F}}_{\sigma (s)} \right]&\le C\, \delta ^{-(1-\varepsilon )p/2}\, y_{\sigma (s)}^{-(1-\varepsilon )p/2} \left( 1+\frac{x_{\sigma (s)}^2}{y_{\sigma (s)}^2}\right) ^{l\varepsilon p/2} \nonumber \\&\le C\, \delta ^{-(1-\varepsilon )p/2}\, e^{-(1-\varepsilon )pas/2} \left( 1+\frac{x_{\sigma (s)}^2}{y_{\sigma (s)}^2}\right) ^{l\varepsilon p/2} \end{aligned}$$

where on the set $A_n$ we have

$$\begin{aligned} 1+\frac{x_{\sigma (s)}^2}{y_{\sigma (s)}^2} \le e^{2n}. \end{aligned}$$

Proceeding from (26), we get from (32) and (27)

33 $$\begin{aligned}&{\mathbb {E}}\left[ 1_{A_n} 1_{\sigma (s) \le t/\delta ^2} |B_{\sigma (s)}|^p \frac{1}{y_{\sigma (s)}^{p/2}} {\mathbb {E}}\left[ |h_{{\sigma (s)},t/\delta ^2}'(z_{\sigma (s)})|^{p/2} \mid {\mathcal {F}}_{\sigma (s)} \right] \right] \nonumber \\&\quad \le C\, {\mathbb {E}}\left[ 1_{A_n} 1_{\sigma (s) \le t/\delta ^2}\, |B_{\sigma (s)}|^p\, e^{-pas/2}\, \delta ^{-(1-\varepsilon )p/2}\, e^{-(1-\varepsilon )pas/2} e^{nl\varepsilon p} \right] \nonumber \\&\quad \le C\, \delta ^{-(1-\varepsilon )p/2}\, e^{nl\varepsilon p}\, e^{-pas+\varepsilon pas/2}\, {\mathbb {P}}(A_n \cap \{\sigma (s) \le t/\delta ^2\})^{1-\varepsilon }\, e^{np} e^{pas} \nonumber \\&\quad = C\, \delta ^{-(1-\varepsilon )p/2}\, e^{np+nl\varepsilon p}\, e^{\varepsilon pas/2}\, {\mathbb {P}}(A_n \cap \{\sigma (s) \le t/\delta ^2\})^{1-\varepsilon }. \end{aligned}$$

We would like to sum this expression in *n*.

#### Proposition 5.6

Let $\sigma (s)$ and $A_n$ be defined as above. Then

$$\begin{aligned}&\sum _{n \in {\mathbb {N}}} e^{np+nl\varepsilon p}\, {\mathbb {P}}(A_n \cap \{\sigma (s) \le t/\delta ^2\})^{1-\varepsilon } \\&\quad \le {\left\{ \begin{array}{ll} C &{}\quad \text {if } p+l\varepsilon p-2r_c(1-\varepsilon ) < 0\\ C(e^{-as}\sqrt{t}/\delta )^{p+l\varepsilon p-2r_c(1-\varepsilon )} &{}\quad \text {if } p+l\varepsilon p-2r_c(1-\varepsilon ) > 0 \end{array}\right. } \end{aligned}$$

where $C < \infty$ depends on $\kappa _-$, $\kappa _+$, *p*, and $\varepsilon$.

#### Proof

We distinguish two cases. If $n \le {\log (\sqrt{t}/\delta )-as+1+a}$, we have [by (25)]

$$\begin{aligned}&\sum _{n \le \log (\sqrt{t}/\delta )-as+1+a} e^{np+nl\varepsilon p}\, {\mathbb {P}}(A_n)^{1-\varepsilon } \\&\quad \le C \sum _{n \le \log (\sqrt{t}/\delta )-as+1+a} e^{np+nl\varepsilon p} e^{-2nr_c(1-\varepsilon )} \\&\quad \le {\left\{ \begin{array}{ll} C &{}\quad \text {if } p+l\varepsilon p-2r_c(1-\varepsilon ) < 0\\ C(e^{-as}\sqrt{t}/\delta )^{p+l\varepsilon p-2r_c(1-\varepsilon )} &{}\quad \text {if } p+l\varepsilon p-2r_c(1-\varepsilon ) > 0. \end{array}\right. } \end{aligned}$$

For $n > {\log (\sqrt{t}/\delta )-as+1+a}$, we have $e^{2(n-1)}e^{2as} > t/\delta ^2$ and therefore (by the definition of $A_n$)

$$\begin{aligned} A_n \cap \{\sigma (s) \le t/\delta ^2\}&\subseteq E_{e^{n-1}}^c \cap \{\sigma (s) \le t/\delta ^2\} \\&\subseteq \left\{ \sigma (s) \le t/\delta ^2 \text { and } 1+\frac{x_{\sigma (s)}^2}{y_{\sigma (s)}^2} > e^{2(n-1)} \right\} , \end{aligned}$$

so Lemma 5.4, applied to $u=e^{-as}\sqrt{t}/\delta$ and $k=2(n-1)-2(\log (\sqrt{t}/\delta )-as)$, implies

$$\begin{aligned} {\mathbb {P}}(A_n \cap \{\sigma (s) \le t/\delta ^2\})&\le C\, (e^{-as}\sqrt{t}/\delta )^{-2r_c}\, e^{-\alpha (2(n-1)-2(\log (\sqrt{t}/\delta )-as)-2a)^2}\\&= C\, (e^{-as}\sqrt{t}/\delta )^{-2r_c}\, e^{-2\alpha (n-(\log (\sqrt{t}/\delta )-as+1+a))^2}. \end{aligned}$$

Consequently,

$$\begin{aligned}&\sum _{n > \log (\sqrt{t}/\delta )-as+1+a} e^{np+nl\varepsilon p}\, {\mathbb {P}}( A_n \cap \{\sigma (s) \le t/\delta ^2\} )^{1-\varepsilon }\\&\quad \le C (e^{-as}\sqrt{t}/\delta )^{p+l\varepsilon p} \sum _{n \in {\mathbb {N}}} e^{np+nl\varepsilon p}\, (e^{-as}\sqrt{t}/\delta )^{-2r_c(1-\varepsilon )}\, e^{-2\alpha (1-\varepsilon ) n^2} \\&\quad \le C (e^{-as}\sqrt{t}/\delta )^{p+l\varepsilon p-2r_c(1-\varepsilon )}. \end{aligned}$$

$$\square $$

Hence, by (33) and Proposition 5.6,

34 $$\begin{aligned}&{\mathbb {E}}\left[ 1_{\sigma (s) \le t/\delta ^2} |B_{\sigma (s)}|^p \frac{1}{y_{\sigma (s)}^{p/2}} |h_{{\sigma (s)},t/\delta ^2}'(z_{\sigma (s)})|^{p/2} \right] \nonumber \\&\quad = \sum _{n=0}^\infty {\mathbb {E}}\left[ 1_{A_n} 1_{\sigma (s) \le t/\delta ^2} |B_{\sigma (s)}|^p \frac{1}{y_{\sigma (s)}^{p/2}} |h_{{\sigma (s)},t/\delta ^2}'(z_{\sigma (s)})|^{p/2} \right] \nonumber \\&\quad \le {\left\{ \begin{array}{ll} C\, \delta ^{-(1-\varepsilon )p/2}\, e^{\varepsilon pas/2} &{}\quad \text {if } p+l\varepsilon p-2r_c(1-\varepsilon ) < 0\\ C\, \delta ^{-(1-\varepsilon )p/2}\, (e^{-as}\sqrt{t}/ \delta )^{p+l\varepsilon p-2r_c(1-\varepsilon )}\, e^{\varepsilon pas/2} &{}\quad \text {if } p+l\varepsilon p-2r_c(1-\varepsilon ) > 0. \end{array}\right. } \end{aligned}$$

Finally, if $p+l\varepsilon p-2r_c(1-\varepsilon ) < 0$, we estimate (24) with (34), so

$$\begin{aligned}&{\mathbb {E}}\left| \delta ^{1/2} \int _0^{\sigma ^{-1}(t/\delta ^2)} |B_{\sigma (s)}| \frac{1}{y_{\sigma (s)}^{1/2}} |h_{{\sigma (s)},t/\delta ^2}'(z_{\sigma (s)})|^{1/2} \, ds \right| ^p\\&\quad \le \delta ^{p/2} \left( \int _0^{\frac{1}{a}\log \frac{C}{\delta }} \left( {\mathbb {E}}\left[ 1_{\sigma (s) \le t/\delta ^2} |B_{\sigma (s)}|^p \frac{1}{y_{\sigma (s)}^{p/2}} |h_{{\sigma (s)},t/\delta ^2}'(z_{\sigma (s)})|^{p/2} \right] \right) ^{1/p} \, ds \right) ^p\\&\quad \le C \delta ^{p/2} \left( \int _0^{\frac{1}{a}\log \frac{C}{\delta }} \left( \delta ^{-(1-\varepsilon )p/2}\, e^{\varepsilon pas/2} \right) ^{1/p} \, ds \right) ^p\\&\quad = C \delta ^{\varepsilon p/2} \left( \int _0^{\frac{1}{a}\log \frac{C}{\delta }} e^{\varepsilon as/2} \, ds \right) ^p\\&\quad \le C. \end{aligned}$$

Since $\varepsilon > 0$ can be chosen as small as we want, the condition to apply this is $p < 2r_c = 1+\frac{8}{\kappa }$.

On the other hand, if $p+l\varepsilon p-2r_c(1-\varepsilon ) > 0$, we have

$$\begin{aligned}&{\mathbb {E}}\left| \delta ^{1/2} \int _0^{\sigma ^{-1}(t/\delta ^2)} |B_{\sigma (s)}| \frac{1}{y_{\sigma (s)}^{1/2}} |h_{{\sigma (s)},t/\delta ^2}'(z_{\sigma (s)})|^{1/2} \, ds \right| ^p\\&\quad \le C \delta ^{p/2} \left( \int _0^{\frac{1}{a}\log \frac{C}{\delta }} \left( \delta ^{-(1-\varepsilon )p/2}\, (e^{-as}\sqrt{t}/\delta )^{p+l\varepsilon p-2r_c(1-\varepsilon )}\, e^{\varepsilon pas/2} \right) ^{1/p} \, ds \right) ^p\\&\quad \le C \delta ^{\varepsilon p/2-(p+l\varepsilon p-2r_c(1-\varepsilon ))} \left( \int _0^{\frac{1}{a}\log \frac{C}{\delta }} e^{as(\varepsilon /2-(1+l\varepsilon -2r_c(1-\varepsilon )/p))} \, ds \right) ^p\\&\quad \le {\left\{ \begin{array}{ll} C &{}\quad \text {if } \varepsilon /2-(1+l\varepsilon -2r_c(1-\varepsilon )/p)> 0\\ C \delta ^{\varepsilon p/2-(p+l\varepsilon p-2r_c(1-\varepsilon ))} &{}\quad \text {if } \varepsilon /2-(1+l\varepsilon -2r_c(1-\varepsilon )/p)< 0 \end{array}\right. }\\&\quad ={\left\{ \begin{array}{ll} C &{}\quad \text {if } 2r_c(1-\varepsilon ) - p(1+\varepsilon (l-1/2)) > 0\\ C \delta ^{2r_c(1-\varepsilon ) - p(1+\varepsilon (l-1/2))} &{}\quad \text {if } 2r_c(1-\varepsilon ) - p(1+\varepsilon (l-1/2)) < 0. \end{array}\right. } \end{aligned}$$

Since $\varepsilon > 0$ can be chosen as small as we want, the condition to apply this is $p > 2r_c = 1+\frac{8}{\kappa }$, and the exponent can be chosen to be greater than $2r_c-p-\varepsilon '$ for any $\varepsilon ' > 0$.

With this estimate for (24), the proof of Proposition 3.5 is complete.

## Footnotes

1 A slightly different result still holds if $\frac{\beta _{1j}-2}{q_{1j}} \ge \theta _j$, as one can see in the proof.

2 Note that in [5], $\lambda$ was called *q*.

3 Note that in [9], the notation $a=2/\kappa$ and $q=r_c-r$ is used.

4 Alternatively, we could also use the same strategy as in the proof of Theorem 2.8, and deduce the result directly from Lemma 2.1.

5 Alternatively, we can drop this condition if we make statements about the SLE$_\kappa$ process only on $t \in [\varepsilon , 1]$ for some $\varepsilon > 0$.

6 Actually, there is only one component because it will turn out that ${\tilde{\gamma }}(\cdot ,\kappa )$ is a simple trace.
